# Stromal cells in the tumor microenvironment: accomplices of tumor progression?

**DOI:** 10.1038/s41419-023-06110-6

**Published:** 2023-09-04

**Authors:** Yan Zhao, Meili Shen, Liangqiang Wu, Haiqin Yang, Yixuan Yao, Qingbiao Yang, Jianshi Du, Linlin Liu, Yapeng Li, Yuansong Bai

**Affiliations:** 1 Department of Oncology and Hematology, China-Japan Union Hospital of Jilin University, 130033 Changchun, Jilin China; 2 Department of Radiation Oncology, China-Japan Union Hospital of Jilin University, 130033 Changchun, Jilin China; 3 Key Laboratory of Special Engineering Plastics Ministry of Education, College of Chemistry, Jilin University, 130012 Changchun, Jilin China; 4 Key Laboratory of Lymphatic Surgery Jilin Province, Jilin Engineering Laboratory for Lymphatic Surgery Jilin Province, China-Japan Union Hospital of Jilin University, 130033 Changchun, Jilin China

**Keywords:** Cancer microenvironment, Cell biology

## Abstract

The tumor microenvironment (TME) is made up of cells and extracellular matrix (non-cellular component), and cellular components include cancer cells and non-malignant cells such as immune cells and stromal cells. These three types of cells establish complex signals in the body and further influence tumor genesis, development, metastasis and participate in resistance to anti-tumor therapy. It has attracted scholars to study immune cells in TME due to the significant efficacy of immune checkpoint inhibitors (ICI) and chimeric antigen receptor T (CAR-T) in solid tumors and hematologic tumors. After more than 10 years of efforts, the role of immune cells in TME and the strategy of treating tumors based on immune cells have developed rapidly. Moreover, ICI have been recommended by guidelines as first- or second-line treatment strategies in a variety of tumors. At the same time, stromal cells is another major class of cellular components in TME, which also play a very important role in tumor metabolism, growth, metastasis, immune evasion and treatment resistance. Stromal cells can be recruited from neighboring non-cancerous host stromal cells and can also be formed by transdifferentiation from stromal cells to stromal cells or from tumor cells to stromal cells. Moreover, they participate in tumor genesis, development and drug resistance by secreting various factors and exosomes, participating in tumor angiogenesis and tumor metabolism, regulating the immune response in TME and extracellular matrix. However, with the deepening understanding of stromal cells, people found that stromal cells not only have the effect of promoting tumor but also can inhibit tumor in some cases. In this review, we will introduce the origin of stromal cells in TME as well as the role and specific mechanism of stromal cells in tumorigenesis and tumor development and strategies for treatment of tumors based on stromal cells. We will focus on tumor-associated fibroblasts (CAFs), mesenchymal stem cells (MSCs), tumor-associated adipocytes (CAAs), tumor endothelial cells (TECs) and pericytes (PCs) in stromal cells.

## Facts

- The TME is made up of cells and extracellular matrix (non-cellular component), and cellular components include cancer cells and non-malignant cells such as immune cells and stromal cells.
- Stromal cells is a major class of cellular components in TME, which also play a very important role in tumor metabolism, growth, metastasis, immune evasion and treatment resistance.
- It is possible to target tumor-associated stromal cell (TASCs) to treat tumors based on the influence of TASCs on tumorigenesis and development.

## Open Questions

- So far, no specific markers have been found in stromal cells in TASC, and finding TASC-specific markers will be more helpful in identifying TASC and targeting TASC for tumor treatment.
- TASCs such as CAFs can be divided into multiple subtypes according to different methods, however, the understanding of CAF subtypes mainly comes from the different classifications of individual experimental teams, and objective and consistent knowledge of their subtypes is required.
- In addition to anti-angiogenic drugs targeting TECs, other strategies targeting stromal cells for tumor therapy have limited efficacy, and how to improve efficacy needs to be further explored.

## Introduction

**Fig. 1:**
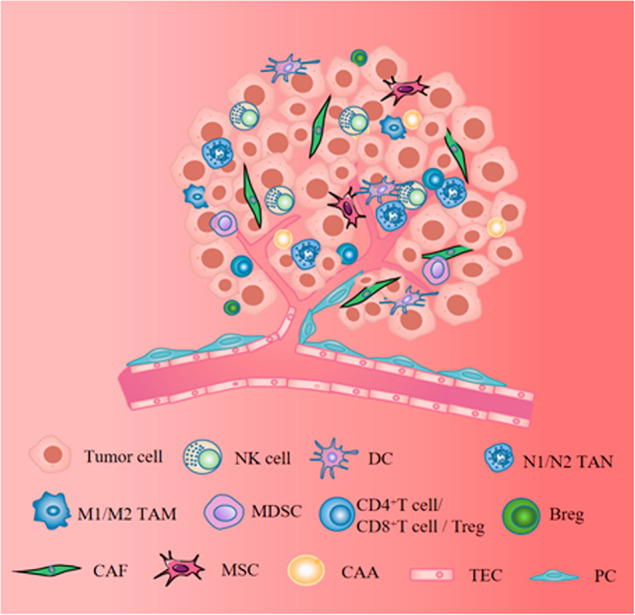
The cellular components of solid tumors are composed of tumor cells, immune cells, and stromal cells, among which immune cells include: DC, NK, TAM, TAN, MDSC, CD4^+^T cell/ CD8^+^T cell/Treg, and Breg. Stromal cells include CAF, MSC, CAA, TEC, and PC. These three types of cells establish complex signaling pathways that affect tumor occurrence, tumor progression, and tumor resistance to treatment. Dendritic cell: DC; Natural killer cell: NK; M1/M2 tumor-associated macrophage: M1/M2 TAM; N1/N2 tumor-associated neutrophil: N1/N2 TAN; Myeloid-derived suppressor cell: MDSC; CD4^+^T lymphocyte/CD8^+^T lymphocyte/regulatory T cell: CD4^+^T cell/CD8^+^T cell/Treg; Regulatory B cell: Breg; Tumor-associated fibroblast: CAF; Mesenchymal stem cell: MSC; Tumor-associated adipocyte: CAA; Tumor endothelial cell: TECs; Pericyte: PC.

Solid tumors are composed of tumor cells and their ecosystem (namely the tumor microenvironment TME) which include various cells (immune cells, stromal cells) and extracellular matrix (ECM), blood vessels, lymphatic vessels, cytokines, mediators, and other non-cellular components [1, 2]. Complex signalings are established among tumor cells, immune cells, and stromal cells in TME thereby influencing tumor genesis, progression, and different clinical outcomes [3]. The previous strategies for treating tumors mostly targeted the cancer cells themselves such as chemotherapy, radiotherapy, and targeted therapy, but the importance of immune cells is gradually recognized in anti-tumor therapy with the discovery of immune checkpoints [4]. Strategies to target immune cells to treat tumors have seen significant clinical efficacy and are recommended by corresponding guidelines, such as T cell-based immune checkpoint inhibitors programmed cell death 1/programmed death-ligand1 (PD-1/PD-L1) antibodies, cytotoxic T lymphocyte-associated protein-4 (CTLA-4) antibodies and chimeric antigen receptor T (CAR-T) in adoptive cell therapy (ACT). Furthermore, the strategy of targeting immune cells to treat tumors also including dendritic (DC) vaccines, reduction of M2 tumor-associated macrophages (TAMs), N2 tumor-associated neutrophils (TANs), bone marrow-derived suppressor cells (MDSCs), regulatory T cells (Tregs), regulatory B cells (Bregs) and the reprogramming of TAMs and TANs into tumor killer cells, etc, and which have been introduced in another article we published [5], therefore, this part of the content will not be covered in this article. In addition, stromal cells, also present in TME (Fig. 1), have also been shown to play a crucial role in tumorigenesis, development, metastasis, and treatment resistance [6]. Each TASC in the tumor microenvironment can communicate with microenvironment components through cytokines and mediators in a paracrine manner or cell-cell interaction, thereby promoting tumor invasion, metastasis, angiogenesis, drug resistance, and recurrence [7, 8]. Therefore, cancer treatment strategies that do not consider stromal cells are not enough. Moreover, understanding the physiological role of each component is essential to understand how they affect tumor behavior and also provide ideas for finding ways to treat tumors based on stromal cells. So this article will focus on TASCs, including the origin of TASCs, the influence of stromal cells on tumorigenesis and development, the mechanism of stromal cell influence on tumors, and strategies for tumor treatment based on stromal cells. The TASCs we will highlight include tumor-associated fibroblasts (CAFs), mesenchymal stem cells (MSCs), tumor-associated adipocytes (CAAs), tumor endothelial cells (TECs), and pericytes (PCs).

### Cancer-associated fibroblasts

CAFs are the most common component of the tumor stroma, especially in the interstitium of breast cancer, prostate cancer, pancreatic cancer, and induration gastric cancer [9–12]. CAFs in TME exhibit two phenotypes: promoting tumor phenotype and inhibiting tumor phenotype, and the former phenotype can participate in tumor genesis, development, and resistance to anti-tumor therapy through multiple mechanisms [13], and this phenotype represents most CAFs groups. Based on this, targeting CAFs has become a strategy for the treatment of tumors, including direct targeting of CAFs and indirect targeting of CAFs through other therapies. In this section, we will introduce the source and classification of CAFs, the relationship with tumors, the mechanism of tumor promotion, resistance to tumor treatment, and targeted CAFs for tumor treatment.

### Sources and types of cancer-associated fibroblasts

CAFs can be formed by attracting fibroblasts of adjacent tissues through transforming growth factor-β (TGF-β), platelet-derived growth factor (PDGF), Fibroblast Growth Factor-2 (FGF-2), and exosomes secreted by tumor cells [14–18]. In addition, CAFs can also be formed by transdifferentiation of normal fibroblasts and TASCs (such as MSCs, TECs, CAAs, and PCs) in tumor tissue [15, 18–23]. For example, up to 80% of normal fibroblasts in breast tissue acquire CAFs phenotype during tumor progression [24]. CAFs are a different cell type from normal fibroblasts, such as mature fibroblasts exhibit a thin, wavy, and small spindle-shaped morphology, while CAFs are often described as immature fibroblasts and appear as large, plump spindle-shaped cells with prominent nucleoli. Moreover, CAFs exhibit dysregulation of signaling pathways and changes in protein expression compared to normal fibroblasts. Dysregulated signaling pathways include up-regulation of TGF-β, bone morphogenetic protein (BMP), Wnt, Sonic hedgehog (Shh), PDGF, Chemotokine (C-X-C motif) ligand 12 (CXCL12)/Chemotokine receptor (CXCR) 4, and integrin-mediated signaling. Changes in protein expression include up-regulation of α smooth muscle actin (α-SMA), fibroblast activating protein (FAP), fibroblast-specific protein-1 (FSP1), platelet-derived growth factor receptor-α (PDGFR-α), PDGFR-β, transcription factor Forkhead box F1 (FOXF1), wave protein, chondroitin sulfate proteoglycan glial antigen-2 (NG2), proline-4-hydroxylase, podophyllotyllin (PDPN), microfibrillary associated protein 5 (MFAP5), collagen 11-α1 (COL11A1) and interstitial matrix(IM) collagens [25, 26]. Not only that, CAFs can produce more collagen types III (PRO-C3) and VI (PRO-C6) than normal fibroblasts [27].

Normal fibroblasts are usually inhibit tumor formation while CAFs exhibit both promoting tumor phenotype and inhibiting tumor phenotype, and the former represents the majority of CAFs and involves in tumorigenesis, development, and resistance to treatment, while the inhibiting tumor phenotype can inhibit tumor proliferation and growth [13, 28]. For example, CAFs in pancreatic cancer are divided into inhibiting tumors subsets and promoting tumors subsets according to their different effects on tumors. Among them, the subsets of CAFs that inhibit tumors include Myofibroblastic (myCAFs), Meflin^+^CAFs, CD271^+^/NGFR^+^CAFs, Gli1^+^CAFs. Tumor-promoting CAFs subsets include Inflammatory CAFs (iCAFs), Zinc finger E-box binding homeobox 1 (Zeb1)^+^CAFs, leucine-rich replication 15^+^ (LRRC15^+^) CAFs, Serum amyloid A3^+^CAFs (SAA3^+^CAFs), FAP^+^/CXCL12^+^CAFs, CD10^+^/GPR77^+^CAFs, CD105^+^CAFs, Hypoxia^+^CAFs, Metabolic CAFs, Antigen-presenting CAFs (apCAFs) [29]. For example, myCAFs have a tumor inhibitory effect, which is located near tumor cells, and the collagen and ECM secreted by myCAFs have an important protective effect in pancreatic ductal adenocarcinoma (PDAC), while the deletion of myCAFs can reduce type I collagen content and significantly reduce tumor tissue hardness thereby leading to aggressive tumors and reducing animal survival rate [29]. Meflin^+^CAFs subsets also have a cancer-suppressing effect, and infiltration of Meflin^+^CAFs can inhibit the growth of xenograft tumors and is associated with a good prognosis for patients, while Meflin-deficient tumor tissue has poor differentiation and tumor progression is significant [6]. In the subset of tumor-promoting CAFs, iCAFs can participate in immune escape or directly act on pancreatic cancer cells by producing inflammatory cytokines such as IL-6, leukemia inhibitor factor (LIF), and CXCL1 to promote tumor progression [30]. CD105^+^CAFs can also promote tumor growth, while CD105^-^CAFs have anti-tumor immunity and tumor suppressor effects [31]. Furthermore, CAFs not only promote tumor growth in mouse models of pancreatic cancer by inhibiting CD8^+^T cells function [32], but also enhance the migration and invasion of cancer cells by inducing β-catenin expression and its nuclear localization [33]. In addition to the presence of CAFs subsets that have a clear effect on tumors, some new CAFs subsets are constantly being discovered. For example, complement secretory CAFs (csCAFs) express many components of the complement system [34], which may play a role in modulating immune and inflammatory responses, but further study is needed. Subtypes of unknown functions also include HoxB6^+^ CAFs. That is, CAFs are not a single cellular entity, but exist as distinct subpopulations and influence tumor biology at several levels.

In addition, CAFs in different tumors can also be divided into different subsets according to different methods, and different subsets have different functions. For example, CAFs can be divided into four different subgroups according to gene expression levels in breast cancer, namely CAF-S1, CAF-S2, CAF-S3, and CAF-S4, of which CAF-S1 can promote the immunosuppressive environment through multiple steps and mechanisms, and CAF-S4 can induce cancer cell invasion through the NOTCH signaling pathway [35]. Not only that the application of single-cell RNA sequencing can identify four subtypes of CAFs with different properties in gastric cancer, namely: myofibroblasts, pericytes, extracellular matrix CAFs (eCAFs), and iCAFs, in which iCAFs can attract and regulate the function of T cells by secreting Interleukin (IL)-6 and chemotokine ligand (CXCL) 12, while eCAFs can reshape ECM in TME and which can form a metastasis-friendly niche by degrading ECM and attracting M2 TAM, moreover, iCAFs and eCAFs not only exhibit enhanced pre-invasive activity but also mobilize surrounding immune cells to build a microenvironment conducive to tumor growth [36]. In addition, the single-cell RNA sequencing of stromal cells in human tumor samples showed that the CAFs subgroup had different transcriptional profiles, the first is mycofabric CAFs which are characterized by high expression of α-SMA and are located near tumor cell nests, followed by iCAFs which are located in fibroproliferative regions away from tumor cells and characterized by low expression of α-SMA and highly expression of IL-6, Leukemia Inhibitory Factor (LIF), IL-11, CXCL1, CXCL8 and platelet-derived growth factor receptor (PDGFR)-α [37]. In short, CAFs can be divided into different subgroups according to different methods, and different subsets have different phenotypes and functions, but in terms of the impact on tumors, CAFs are mainly divided into two subtypes: pro-tumor and inhibition of tumors, and in the next section we will introduce in detail the relationship between CAFs and tumors.

### Cancer-associated fibroblasts and tumors

The CAFs in TME can be divided into different subtypes according to different methods, and different subtypes have differences in protein expression, paracrine signaling, tumorigenicity, and aggressiveness [26]. However, most of the CAFs population in TME have a pro-tumor phenotype and play a promoting role in the occurrence and development of tumors. For example, in breast cancer, CAFs can promote the metastasis of precancerous and malignant breast epithelial cells while normal fibroblasts promote epithelioid phenotype and inhibit metastasis [38]. Similarly, normal prostate epithelial cells cause intraepithelial neoplasia when co-injected with CAFs but not when co-injected with normal fibroblasts [39]. CAFs can also trigger nonmalignant cell malignant transformation through overexpression of estrogen, TGF-β, and Hepatocyte Growth Factor (HGF) [40, 41]. In conclusion, CAFs are involved in tumorigenesis.

CAFs are not only involved in tumorigenesis, but also play a very important role in tumor metastasis. For example, the migration potential of lung cancer cell have increased when treated with CAFs culture medium compared with normal fibroblast culture medium [42]. Co-transplantation of cervical cancer cells with CAFs into mice results in lymph node metastasis while injection without CAFs does not [43]. Moreover, CAFs can also participate in the formation of ecological niche before lung metastasis by secreting exosomes [44]. In addition, CAFs are also closely related to tumor invasion and progression [19], such as the Hyaluronan and proteoglycan link protein 1 (HAPLN1) derived from CAFs can promote tumor invasion [45]. CAFs with high expression of FOS-like antigen 2 (FOSL2) can promote angiogenesis and tumor growth [46].

In addition, IL-6 and IL-8 released by CAFs not only promote cancer cell invasion but also participate in tumor angiogenesis [47]. Conversely, blocking the IL-6/JAK2/STAT3 pathway can inhibit the progression of precancerous vocal cord (oral) leukoplakia and delay the occurrence of head and neck squamous cell carcinoma (HNSCC) tumors [48]. In conclusion, CAFs are involved in tumor invasion and metastasis.

In addition, CAFs are similar to circulating tumor cells (CTCs), such as circulating CAFs can be detected in the blood and may be related to tumor progression. For example, circulating CAFs can be detected in the blood of both breast cancer and prostate cancer with high levels of CAFs, and circulating CAFs exist in 88% of metastatic breast cancer patients and 23% of non metastatic patients (based on the expression of FAP and actin alpha 2 ACTA2) [49]. Similarly, circulating CAFs (the expression of waveform protein is positive, the expression of cytokeratin is negative) are also found in 58% of patients with metastatic prostate cancer but not in patients with nonmetastatic disease [50]. That is to say, circulating CAFs may be related to tumor progression. In short, CAFs play a very important role in the occurrence, development, and metastasis of tumors, which makes it particularly important to explore the tumor-promoting mechanism of CAFs.

### Protumor mechanisms of cancer-associated fibroblasts

**Fig. 2:**
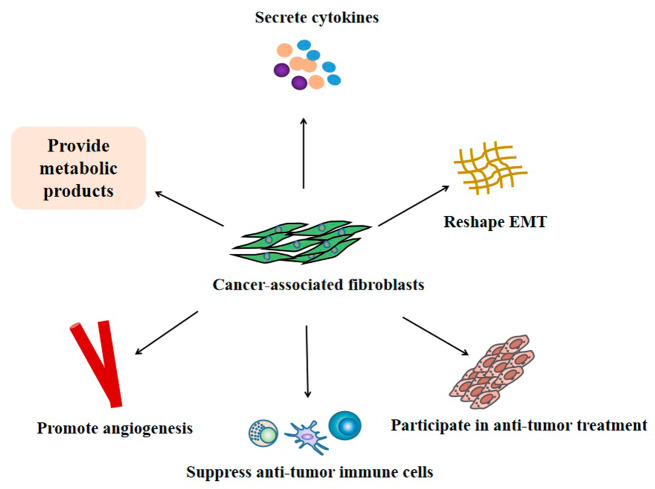
The mechanism of cancer-associated fibroblasts (CAFs) promoting tumor growth. CAFs can promote tumor growth through a variety of mechanisms: such as secreting a variety of cytokines; reshaping the extracellular matrix (ECM); promoting angiogenesis; inhibiting anti-tumor immune cells, providing metabolites (such as lactic acid, amino acids, fatty acids) to tumor cells; and participating in resistance to anti-tumor treatment, etc.

CAFs can promote tumorigenesis, invasion, metastasis, and resistance to treatment through a variety of mechanisms (Fig. 2) [51]. For example, compared with normal tissue fibroblasts, CAFs can increase tumor cell proliferation, ECM production, and also promote the secretion of various cytokines (such as stromal cell-derived factor-1 SDF-1; Vascular endothelial growth factor VEGF; HGF [46, 52–55]. Such as CAFs can reshape the ECM to provide a supportive microenvironment for cancer cells and become a physical barrier for drug penetration [56, 57]. In addition, CAFs can also secrete matrix metalloproteinases (MMPs) to interfere with the degradation of ECM, and initiate the migration and invasion of cancer cells in the process of promoting and degrading ECM [58].

CAFs can also promote tumor occurrence, invasion, angiogenesis, and metastasis by secreting various factors [7, 59], in addition to influence tumor growth and therapeutic effect by adjusting the structure of ECM. For example, CAFs and cancer cells communicate bidirectionally through secreting of inflammatory cytokines such as IL-1A, IL-1B and Tumor Necrosis Factor (TNF) [60]. Moreover, IL-6 secreted by CAFs has a tumor-promoting effect in highly invasive tumors [61]. CXCL14 and Chemotokine ligand (CCL)2 secreted by CAFs can promote tumor cell survival, growth, and mediate Epithelial-mesenchymal transformation (EMT) [62, 63]. At the same time, α-SMA, integrin α11 and PDGF-BB secreted by CAFs can enhance the invasion potential of cancer cells [64, 65]. Moreover, the SDF-1 and PDGFC secreted by CAFs can promote cancer progression, metastasis, and angiogenesis [66–68]. In addition, Chitinase 3-like 1 (CHI3L1) secreted by CAFs acts on CAFs can increase the secretion of IL-8 and affect the angiogenesis of colorectal cancer tumors [69]. Simultaneously, CAFs express and secrete Collagen and calcium-binding EGF domain-1 (CCBE1) thereby promoting VEGFC maturation and lymphangiogenesis in colorectal cancer [70]. In short, CAFs can promote tumor genesis, invasion and metastasis by secreting various factors.

On the other hand, various factors secreted by CAFs can also promote tumor progression by regulating immune cells in TME [28]. For example, CAFs can inhibit natural killer (NK) cell function and the expression of NK receptors, perforin, and granzyme B by secreting cytokines, chemokines, and MMPs [71]. Moreover, CAFs can promote the recruitment of monocytes and promote their transformation into M2 TAMs by releasing IL-8, IL-10, TGF-β, and CXCL12 [28, 72, 73]. CAFs can also recruit MDSCs, and activate monocyte MDSCs (M-MDSCs) through secreting CCL2 and IL-6 by CAFs and miR-21 derived from CAFs, and further activate the production of MDSCs [74–76]. In addition, CAF-derived cardiotrophic factor-like cytokine 1 (CLCF1) can promote the infiltration and polarization of TANs in a paracrine manner [2]. Not only that, CAFs can also limit the intratumoral infiltration of CD8^+^ T cells and induce the transformation of primitive CD4^+^T cells into Tregs [77, 78]. To sum up, various factors secreted by CAFs can promote tumor progression by regulating immune cells in TME to form an immunosuppressive microenvironment.

At the same time, exosomes derived from CAFs are also involved in tumor genesis and development. For example, CAFs can promote angiogenesis of cancer cells through the miR-135b-5p/FOXO1 axi. In addition, the downregulation of miR-214 expression in CAFs can promote cancer cell EMT in tumors [79, 80], while the expression of miR-31 upregulated and miR-1 and miR-206 downregulated can inhibite autophagy of CAFs and promote tumor migration and recruitment of TAMs [81, 82]. In addition, the long non-coding RNA (lncRNA) MIR155HG secreted by CAFs also has carcinogenic effects, which are associated with decreased apoptosis and enhanced cell growth. In addition, CAFs can also initiate the expression of lnc HOTAIR to promote EMT and tumor metastasis [83, 84]. At the same time, exosomes derived from CAFs can also provide tumor cells with mitochondrial genomes to improve tumor oxidative phosphorylation and mitochondrial metabolism and further enhance tumor drug resistance and the self-renewal ability of cancer stem cells (CSCs) [85]. In summary, exosomes derived from CAFs can participate in the occurrence and development of tumors through various mechanisms.

In addition, CAFs can also play a tumor-promoting role by adjusting the metabolism with tumor cells. For example, CAFs participate in and promote tumor metabolic reprogramming through a variety of mechanisms, such as direct/indirect export of nutrients, provision of mitochondria, and regulation of metabolic enzyme activity. For example, the aerobic glycolysis of CAFs in TME increased and futher produce a large number of metabolites pyruvate and lactic acid as a source of nutrition and energy for tumor biosynthesis [86]. CAFs can also provide amino acids needed for tumor cells growth by directly synthesizing glutamine and other amino acids [87]. At the same time, the lipid metabolism of CAFs is also reprogrammed, such as lysophosphatidylcholine (LPCs) are secreted into microenvironment and directly absorbed and utilized by tumor cells to form membrane lipids [88]. In addition, the exosomes released by CAFs also contain complete metabolites including amino acids, lipids, and tricarboxylic acid cycle (TCA) cycle intermediates, and which are widely used by cancer cells for carbon metabolism in times of nutritional deficiency or stress [89]. Moreover, exosomes secreted by CAFs can also reorganize cancer cell metabolism through the enrichment of exosomes non-coding RNA, such as upregulating the glycolytic metabolism of cancer cells [90]. In a word, CAFs can support tumor growth through multiple mechanisms, such as the production of various nutrients and metabolic reprogramming of cancer cells.

### Cancer-associated fibroblasts and tumor-treatment resistance

CAFs not only participate in the occurrence and development of tumors through various mechanisms, but also participate in the resistance of tumors to various anti-tumor treatments such as chemotherapy, radiotherapy, endocrine therapy, targeted therapy, and immunotherapy. For example, the HNSCC cells are insensitive to cisplatin (CDDP) after co-culture with CAFs and HNSCC cells [91], which indicated that tumor drug resistance is closely related to CAFs. The mechanisms by which CAFs cause cancer cells to resist anti-tumor treatment involve a wide range of mechanisms, such as secreting cytokines, exosomes, and expressing different proteins (different CAFs subtypes) to participate in resistance to anti-tumor therapy. The first is the cytokines secreted by CAFs lead to anti-tumor therapy resistance, such as activated CAFs promoting cancer cell resistance to CDDP by secreting IL-6 [46]. CAF-derived IL-8 can promote gastric cancer resistance to chemotherapy by activating NF-κB and upregulating ATP-binding cassette, sub-family B (ABCB1) [92]. Not only that, CAFs can also activate the Wnt/β-catenin pathway in EOC cells (mouse microglia) through the CXCL12/ CXCR 4-axis, resulting in cancer cells resistant to CDDP [93]. In addition, CAFs-derived CCL5 can promote tumor resistance to CDDP by upregulating the expression of lncRNA HOTAIR [94]. Cytokines secreted by CAFs not only participate in tumor resistance to chemotherapy, but also participate in tumor resistance to radiotherapy. For example, CXCL1 secreted by CAFs can mediate radiation resistance by inhibiting the expression of the reactive oxygen species (ROS)-scavenging enzyme superoxide dismutase 1 or by activating the mitogen-activated proteinkinase kinase (MEK)/extracellular regulated protein kinases (ERK) pathway [95]. In addition, the insulin-like growth factor-binding proteins (IGFBP)-2, -4, and -6, insulin growth factor 2 (IFG2), and PDGF-AA produced by CAFs can also mediate tumor resistance to radiotherapy [96]. Moreover, the cytokines produced by CAFs are also involved in tumor resistance to targeted therapies, such as fibroblasts that produce HGF lead to resistance of lung cancer cells to epidermal growth factor receptor tyrosine kinase inhibitors (EGFR-TKIs) [97]. In short, CAFs can participate in the resistance of tumors to anti-tumor treatments such as chemotherapy, radiotherapy, and targeted therapy by secreting various cytokines.

At the same time, CAFs can also mediate tumor resistance to treatment by secreting exosomes. For example, chemotherapy drugs CDDP and paclitaxel inhibit arachidonate lipoxygenase by activating the Ubiquitin Specific Peptidase 7 (USP7)/heterogeneous nuclear ribonucleoprotein A1 (hnRNPA1) axis 15 (ALOX15) and reduce the accumulation of lipid-reactive oxygen species (ROS) in cancer cells and ultimately lead to a decrease in chemotherapy sensitivity [98]. CAFs derived exosomes miR-196a make advanced head and neck cancer resistant to CDDP by targeting cyclindependent kinase inhibitor 1B (CDKN1B) and inhibitor of growth family (ING5) [99]. The exosome LINC00355 also from CAFs can promote the resistance of breast cancer cells to CDDP by regulating the miR-34b-5p/ATP binding cassette, subfamily B (ABCB1) axis [100]. While CAFs-derived exosome microRNA-98-5p (miR-98-5p) can promote CDDP resistance in ovarian cancer by downregulating monoclonal antibody to cyclin-dependent kinase inhibitor 1A (CDKN1A) [101]. Furthermore, exo-miR-103a-3p derived from CAFs can promote CDDP resistance in non-small cell lung cancer (NSCLC) cells [102]. In addition, the lncRNA SNHG12 carried by CAFs-exosomes enters NSCLC cells then promotes RNA stability and X-linked inhibitor of apoptosis protein (XIAP) transcription by binding to HuR, thereby enhancing NSCLC cell resistance to CDDP [103]. Furthermore, exosomes secreted by CAFs are also involved in tumor resistance to radiotherapy in addition to mediating tumor resistance to chemotherapy drugs. For example, exosomes released by CAFs can stimulate Retinoic Acid Inducible Gene 1 Protein (RIG-1) signaling in cancer cells, and Jagged 1 (JAG1) on CAFs can activate NOTCH3 signaling on cancer cells, and these pathways work together to promote tumor resistance to radiation therapy and chemotherapy [104]. In summary, CAFs can mediate tumor resistance to antitumor therapy by secreting multiple exosomes.

In addition, CAFs can also participate in resistance to anti-tumor treatment by expressing different proteins (also known as different subtypes of CAFs). For example, CAFs expressing neuropilin 2 (NRP2) can reduce the sensitivity of gastric cancer cells to 5-fluorouracil (5-FU) [105]. α-SMA(+) CAFs can enhance hepatocellular carcinoma (HCC) resistance to chemotherapy by stimulating the HGF-MET-FRA1-HEY1 cascade reaction [106]. Furthermore, CAFs that express specific proteins are also involved in tumor resistance to targeted therapy, such as CAFs expressing high-level Neuregulin 1 (NRG1) can lead to trastuzumab resistance in HER-2+ breast cancer through the HER3/AKT pathway [107]. Furthermore, CAFs-rich tumors are also not sensitive to combined immune checkpoint inhibitors (ICB) therapy [63], indicating that CAFs are involved in tumor resistance to ICB therapy. For example, ecm-myCAF, TGF-ß- myCAF, and wound myCAF are known drivers of immunosuppressive environment and immune therapy resistance in the CAFs subtype [108], in which ecm-myCAF upregulates the levels of PD-1 and CTLA4 proteins in Tregs, and the pan-CAF subtype expresses immunosuppressive inflammatory factors CXCL12, CXCL14, and stem cell promoting factor IL-6, all of which are related to ICB resistance [109]. In summary, CAFs can not only participate in tumor resistance to treatment by secreting cytokines and exosomes, but also some subtypes of CAFs or CAFs expressing different proteins in CAFs can participate in tumor resistance to tumor treatment.

### Strategies for targeting cancer-associated fibroblasts for tumor therapy

Based on the tumor-promoting effect of most CAFs in TME, the strategy of targeting CAFs as target cells for tumor treatment has become possible, including direct targeting of CAFs and indirect targeting of other treatments that affect CAFs. Direct targeting of CAFs include preventing CAFs infiltration, inhibiting CAFs activation, reducing the number of CAFs, reprogramming CAFs (restoring the phenotype of quiescent fibroblasts or converting CAFs to an inhibitory phenotype), and developing CAFs oriented vaccines and therapies. While indirect targeting of CAFs include radiotherapy and chemotherapy, targeting immune cells, targeting downstream effectors of CAFs, targeting CAFs related signaling pathways, and targeting ECM proteins derived from CAFs, which all can indirectly affect the number and activity of CAFs.

The treatment strategy of directly clearing CAFs mainly relies on the surface markers of CAFs such as FAP, α- SMA and PDGFR [110]. It has been reported that type II membrane-bound glycoprotein FAP is not expressed in normal tissues but expressed in activated CAFs in the tumor stroma, and FAPα-expressing vaccines can inhibit the growth of 4T1 tumors (breast cancer) by killing CAFs by generating FAPα-specific cytotoxic T lymphocyte (CTL) responses [111]. In addition, T cells triggered by DC/CAF fusion cells can also produce a strong CTL immune response to CAFs [112].

In addition to directly targeting CAFs to affect the number and activity of CAFs, other treatments also indirectly affect CAFs. Scriptaid (a small molecule inhibitor of histone deacetylase inhibitor (HDACs) 1/3/8) can inhibit CAFs differentiation and reduce the number of CAFs through TGF-β [113]. The drug pirfenidone (PFD) can reduce the capacity of aggressiveness and immunosuppressive mediated by CAFs in breast cancer [114]. While all-trans-retinoin and minnelide (which de-regulates the TGF-β signaling pathway) can calm the active CAFs [115, 116]. In addition, the vitamin D receptor (VDR) ligand calcipotriol can also reduce the proliferation and migration of CAFs [117], and the treatment of gastric cancer cells with calcipotriol can eliminate CAF-derived IL-8-mediated resistance to platinum oxalate by blocking the PI3K/Akt signaling pathway [118]. Moreover, tocilizumab (the inhibitor of IL-6 receptor) can inhibit the cancer-promoting effect of CAFs in breast cancer by inhibiting the STAT3/AU-rich element RNA-binding protein 1 (AUF1) pathway [114]. Not only that, eicosapentaenoic acid can inhibit angiogenesis through reducing the secretion of IL-6 and VEGF by CAFs in colon cancer [53]. In addition to drugs indirectly affecting the quantity and activity of CAFs, other treatments also indirectly affect CAFs. For example, knockout of shRNA mediated MMP2 gene can reduce the release of ECM fibers from CAFs and prevent lung metastases in breast cancer [119]. Reducing the expression of Cyclooxygenase-2 (COX-2) in cells can reduce the number of CAFs [120]. Furthermore, inhibition of CC chemokine receptor (CCR) 2 and elimination of ROS can eliminate the CAF-MDSC axis, thereby favoring the reversal of CAF-mediated immunosuppressive microenvironment [121]. Moreover, inhibition of ROS-producing enzymes nicotinamide adenine dinucleotide phosphate oxidase 4 (NOX4) expressed on CAFs can promote CD8^+^ T cell infiltration and enhance tumor response to ICB therapy [122]. In addition, cytokines can also affect the quantity and activity of CAFs. For example, TGF-β can downregulate CAFs by binding mothers against DPP homolog 1 (SMAD) to CCBE1 [69]. Interferon (IFN)-γ can inhibit fibroblast-leading tumor cell invasion by inhibiting fibroblast motility and their adhesion to tumor cells [58]. In short, the strategy of treating tumors with CAFs as target cells can be achieved direct action on CAFs or indirect effects such as drugs, cytokines, or reducing the expression of some enzymes.

Targeted CAFs for tumor therapy can be achieved through a variety of strategies. However, it is better to selectively reduce the tumor-promoting CAFs subsets and protect the tumor-suppressing CAFs subsets because there are both tumor-promoting and tumor-suppressing CAFs subsets in TME. For example, Meflin^+^CAFs can inhibit tumors, while the application of unnatural retinol Am80 can effectively induce Meflin expression of CAFs in PDAC, and Am80 administration can not only increase tumor vascular area and intratumor drug delivery, but also improve the sensitivity of PDAC to chemotherapy drugs [123]. While apCAFs are tumor-promoting CAFs subtype and derived from mesothelial cells, antibody therapy targeting the mesothelial marker mesothelin can effectively inhibit the conversion of mesothelial cells to apCAF [78]. Anti-GPR77 antibody injection can significantly reduce the infiltration of CD10^+^GPR77^+^CAFs (pro-tumor subsets of CAFs), and anti-GPR77 antibody combined with chemotherapy can significantly enhance the apoptosis of tumor cells and CAFs, and inhibit tumor growth [124]. FAP^+^CAFs also belong to the pro-tumor CAFs subgroup, which can secrete CXCL12 to inhibit the accumulation of cytotoxic CD8^+^T cells in tumors, while the use of CXCL12 receptor chemokine receptor 4 inhibitor AMD3100 not only causes rapid accumulation of CD8^+^T cells among cancer cells, but also enhances the efficacy of anti-PD-L1 therapy [125]. Furthermore, IL1 can induce LIF expression and activate downstream JAK/STAT to generate tumor-promoting CAFs subtype iCAFs, while TGF-β can antagonize this process by downregulating interleukin-1 receptor1 (IL1R1) expression and promoting differentiation into myCAFs [30]. In addition, the angiotensin II type 1 receptor (AGTR1) was identified as a marker of iCAF, and the inhibitor of this receptor Losartan has been shown to reduce intratumoral solid stress thereby increasing vascular perfusion and improving drug delivery [126]. In addition. diphtheria toxin (DT) can selectively deplete the pro-tumor CAFs subtype LRRC15^+^CAFs, and can also lead to CAFs components being recalibrated towards universal fibroblasts [127]. In short, based on the continuous discovery of CAFs subtypes in TME, reducing the tumor-promoting CAFs subsets in TME and increasing the tumor-inhibiting CAFs subsets can make the targeted CAFs treatment of tumors more accurate.

TME plays a crucial role in the occurrence, development, and metastasis of tumors, among which CAFs as the largest type of stromal cells in TME have been more well understand, including its origin, relationship with tumors, and tumor promoting mechanisms (by releasing cytokines, exosomes, and metabolites to participate in cancer cell growth and affect tumor cell resistance to treatment [128, 129]. Numerous studies have also been carried out on the treatment of CAFs, but most of these studies are in the preclinical stage, and clinical trials on CAFs and tumors mostly focus on tumor PET imaging of FAP (molecular markers of CAFs). There are only 3 clinical trials targeting CAFs for tumor treatment, including the “Phase 1 Dose escalation Trial of OMTX705, an Anti fiber last Activation Protein Antibody dry Conjugate, as Single Agent and in Combination With Pembrolizumab in Patients With Advanced Solid Tumors” (NCT05547321) and “ Phase I/II Investigator-initiated Clinical Trial of MIKE-1 With Gemcitabine and Nab-paclitaxel Combination Therapy for Unresectable Pancreatic Cancer“ (NCT05064618). In the latter clinical trial, MIKE-1 (Am80) is a synthetic unnatural retinoic acid, which can effectively convert Meflin^-^pCAF into Meflin^+^rCAF, and this clinical trial is to combine MIKE-1 with gemcitabine (GEM) and nab-paclitaxel (nab-PTX) in patients with unresectable pancreatic cancer and evaluate the safety, tolerability and efficacy of this treatment. Another clinical trial is “Basket Study to Evaluate the Therapeutic Activity of Simlukafusp Alfa as a Combination Therapy in Participants With Advanced and/or Metastatic Solid Tumors” (NCT03386721), this clinical trial is an open label, multicenter, Phase II study, in which Simlukafusp α is an immune cytokine consisting of an interleukin-2 variant (IL-2V) targeting FAP-α, and this test aims to evaluate the antitumor activity of Simlukafusp α in combination with atezolizumab (anti-PD-L1) in patients with advanced and/or metastatic solid tumors. Although a large number of preclinical studies have been carried out on CAFs for tumor treatment and some clinical trials are also underway, no CAFs-specific inhibitors have been approved so far, which may be related to the lack of specific targets for CAF and the fact that ongoing clinical trials have not yet been completed. However, since CAFs represent most cells in the tumor stroma and most CAFs have tumor-promoting properties, the treatment of tumors against CAFs will become another major and important strategy for the treatment of tumors.

### Mesenchymal stem cells

MSCs are a heterogeneous population of stromal cells present in the interstitium of various tissues and organs and are also localized in various primary and metastases tumors [130–132]. MSCs have different roles in different stages of tumorigenesis, such as it may have the effect of inhibiting tumorigenesis and growth in the early stage of tumors, but promoting tumor development through a variety of mechanisms in the later stage of tumorigenesis [133]. Moreover, MSCs can be used as an ideal drug carrier for the treatment of tumors based on which have the property of tumor homing. We will focus on the source and homing of MSCs, the relationship between MSCs and tumors, and the strategies for treating tumors based on MSCs.

### Sources and homing of mesenchymal stem cells

MSCs are plastic, adherent cells that specifically express CD73, CD90, and CD105, but do not CD34, CD45, CD14, CD11b, CD79a, CD19, and Human leukocyte antigen (HLA)-DR. It can be isolated from various tissues such as bone marrow, adipose tissue, umbilical cord [134], or induced by pluripotent stem cells [135], and can differentiate into adipocytes, osteoblasts, and chondrocytes [136, 137]. Moreover, MSCs can migrate to the tumor site through the interaction of a variety of chemokine receptors (such as CCR-1, CCR-2, CCR-3, CCR-4, CCR-5, CCR-7, CXCR-1, CXCR-2, CXCR-3, CXCR-4) on MSCs and cytokines or chemokines (such as endothelial cell selectin, MMPs, IL-8, PGDF-AB, IGF-1, VEGF) secreted by solid tumors [138–140]. The SDF1 receptor CXCR4 expressed by MSCs and the highly expressed SDF1 on the surface of tumor cells can migrate MSCs to the tumor through the CXCR4-SDF1 axis [141]. The chemokine CCL5 produced by MSCs combines with the homologous receptor CCR5 on human breast cancer cells (BCC) can promote lung metastasis of breast cancer [142]. Meanwhile, studies have also shown that cigarette smoke extract (CSE) and benzo[α]pyrene (B[α]P) can increase osteopontin (OPN) expression levels and promote the recruitment and adhesion of MSCs to lung cancer cells through JAK2/STAT3 signaling [132]. In addition, cytokines TNF-α, IL-6, IL-1β, and IFN-γ are involved in the adhesion of circulating MSCs to the vascular endothelial layer [143], and MSCs adhering to the vascular endothelial layer enter tumor tissue through the vascular wall [134, 140, 144]. Conversely, reducing these factors or receptors can reduce the migration and invasion of MSCs into cancer cells [145]. In conclusion, the interaction between cancer cells and MSCs promotes the homing of MSCs to tumors.

### Mesenchymal stem cells and tumors

As another major class of stromal cells in TME, MSCs have different roles in different stages of tumor development. For example, in the early stage of tumorigenesis or exogenous MSCs have the effect of inhibiting tumorigenesis and growth. Such as co-culture of umbilical cord-derived MSCs with glioblastoma stem cells (CSCs) showed that the proliferation rate of CSCs was significantly reduced [146], and human cord-derived MSCs could also effectively inhibit the growth of liver cancer in mice [147]. Moreover, the systematic administration of allogeneic MSCs neither accelerates the progression of precancerous lesions nor increases the malignancy of precancerous lesions, but rather prevents the progression of oral squamous cell carcinoma (OSCC) and tumor growth [148]. At the same time, donor MSCs can also reprogram host macrophages and restore the bone marrow microenvironment, thereby inhibiting the development of leukemia and prolonging the survival of leukemia mice [149]. Moreover, exosomes secreted by MSCs are also involved in tumor inhibition. Such as exosomes derived from human MSCs can induce tumor cell apoptosis and necrosis in hepatocellular carcinoma, ovarian cancer, and kaposi’s sarcoma by activating negative cell cycle regulatory factors [150]. In addition, exosomes derived from human umbilical cord MSCs have anti-proliferation and pro-apoptosis effects in bladder cancer [151]. Exosomes derived from human adipose MSCs can also inhibit the proliferation of ovarian cancer cells and induce their apoptosis [152]. Furthermore, MSCs can also block tumor cell proliferation in G0/G1 phase of the cell cycle through cell-to-cell contact, thereby preventing tumor cells from entering S phase of the cell cycle [153]. All of the above indicated that MSCs have the effect of inhibiting tumor occurrence and growth. However, MSCs homing to the tumor site in the later stage of tumor development, which are “re-educated” by tumor cells and other cells in TME and make MSCs have the characteristics of promoting tumorigenesis, development and metastasis [19, 154].

**Fig. 3:**
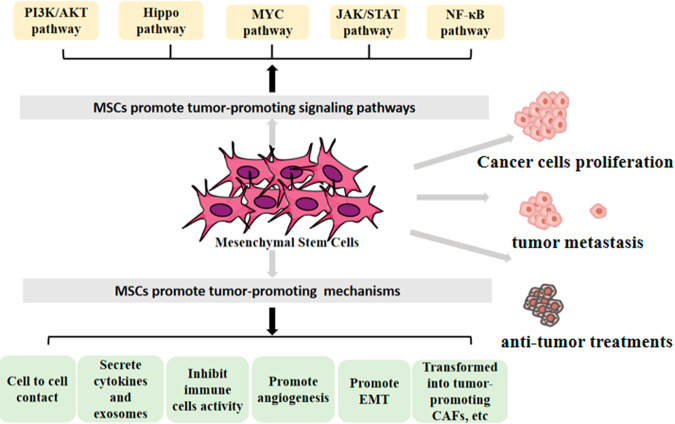
Mesenchymal stem cells (MSCs) promote tumor progression through multiple mechanisms and multiple pathways, among which signaling pathways include: PI3K/AKT pathway, Hippo pathway, MYC pathway, JAK/STAT pathway, NF-κB pathway, etc. The mechanisms include: cell-to-cell contact, secretion of cytokines and exosomes, inhibition of immune cells activity, promotion of angiogenesis, promotion of epithelial interstitial transformation (EMT), transforming into cancer-associated fibroblasts (CAFs), etc, and ultimately promote cancer cells proliferation, tumor metastasis and anti-tumor treatments.

MSCs in TME can promote the occurrence and development of tumors through mechanisms such as cell-to-cell contact, secretion of biomolecules, enhancement of angiogenesis, inhibition of immune cell activity, or conversion to CAFs (Fig. 3). For example, MSCs can be engulfed by cancer cells, and the metastasis and aggressiveness of cancer cells are enhanced after ingestion [152]. MSCs can also enhance tumor vascularization by upregulating VEGF and IL-6 [155], and promote the progression and metastasis of cancer by secreting CCL5, CCL7, and TGF-β [142, 156]. Not only that MSCs also affect anti-tumor immune function, such as inhibiting the antitumor activity of NK and DC cells [157], inducing macrophage M2 polarization [158], promoting Tregs production, and reducing B cell activation [159]. In addition, exosomes derived from MSCs can also participate in immunomodulation by regulating immune cell function and altering the secretion of inflammatory factors (such as TNF-α and IL-1β) [160]. For example, exosomes derived from MSCs can accelerate the progression of breast cancer by inducing monocytes myeloid-derived suppressor cells (M-MDSCs) to differentiate into M2 TAMs in the tumor bed [161]. Meanwhile, other exosomes derived from MSCs can also promote the proliferation, migration, and angiogenesis of cancer cells, such as miR-410, miR-130b-3p, miR-21-5p and miR-15a [162]. In addition, MSCs in TME can also differentiate into CAFs thereby indirectly promoting tumor progression [163]. In short, MSCs homing to the tumor site can promote the occurrence and development of tumors through various mechanisms such as secreting biomolecules, exosomes, and affecting anti-tumor immune function.

The tumor-promoting mechanism of MSCs can be achieved through multiple pathways. Such as MSCs promote tumor generation and progression by regulating and activating the PI3K/AKT signaling pathway and regulating the Hippo pathway [164, 165]. MSCs can also promote gastric cancer growth by upregulating c-Myc [166], and promote the progression of colorectal cancer by activating mTOR and NF-κB signaling [167]. In addition, IL-6 secreted by MSCs can promote tumor progression by activating JAK2/STAT3 signaling and upregulating NF-κB [168, 169]. At the same time, galectin3 expressed on MSCs can promote adhesion between MSCs and acute myeloid leukemia (AML) tumor cells by activating the MYC signaling pathway thereby promoting the survival of cancer cells [170]. In summary, the tumor promoting effect of MSCs can be achieved through multiple pathways.

In addition, MSCs are also involved in tumor resistance to treatment, such as co-culturing human oral squamous cell line JHU-012 cells with bone marrow MSCs can lead to resistance of JHU-012 cells to CDDP [171]. Moreover, bone marrow MSCs can also make SCC-25 cells (human tongue squamous cell carcinoma cells) resistant to paclitaxel by up-regulating BCL-2, POSTN, multiple drug resistance-related protein 1 and human adenosine triphosphate binding cassette transporter G (ABCG transporter) [172]. Moreover, exosomes derived from MSCs are also involved in the resistance of tumors to treatment. For example, MSC-derived exosomes can induce 5-FU resistance in gastric cancer cells by activating the CaM-Ks/Raf/MEK/ERK pathway [173]. In addition, the expression of S100A6 mediated by miR-21-5 in breast cancer can enhance the resistance to doxorubicin [174]. Moreover, human bone marrow microenvironment-derived MSCs can promote the proliferation of chronic myeloid leukemia (CML) cells while reducing their sensitivity to tyrosine kinase inhibitors [175]. That is to say, MSCs are involved in tumor resistance to chemotherapy and targeted therapy.

### Strategies for the treatment of tumors based on mesenchymal stem cells

MSCs are considered to be an ideal carrier for cancer treatment due to their ability to homing tumors. The use of MSCs to treat tumors does not promote tumor progression despite MSCs in TME are involved in tumorigenesis and progression through multiple mechanisms such as intercellular contact, regulation of anti-tumor immunity, secretion factors, and exosomes [154]. This is because MSCs require 4 to 30 days to form a tumor-promoting phenotype (such as MSC conversion to CAF) [133], while most exogenous MSCs disappear from the body within a week due to cell death [176]. That is, endogenous mesenchymal stem cells are involved in tumorigenesis and progression, but the administration of mesenchymal stem cells may have less effect on tumor growth.

This point of view has also been proven by the facts. Such as the use of MSCs from bone marrow, adipose tissue and umbilical cord blood as carriers to deliver anti-cancer cell factors, pro-apoptotic proteins, suicide genes, oncolytic viruses (OVs), and chemotherapy drugs have all shown antitumor effects [143]. For example, the establishment of IFN-α overexpressed MSCs (IFNα-MSCs) not only eliminates tumors in situ, but also has a specific antitumor effect on distant tumors after intratumoral injection [177]. In addition, miR34a-modified MSCs can not only induce glioma cell aging, but also induce DNA damage through regulation of Sirtuin 1 (SIRT1) [178]. Similarly, human placenta-derived MSCs transduced by the HGF antagonist NK4 can inhibit tumor cell growth by inducing apoptosis [179]. At the same time, transgenic MSCs can also be used as carriers of immunomodulatory proteins to deliver them to tumor tissues and can improve the efficacy of CAR-T cells in the treatment of solid malignancies [180]. Not only that, MSCs can also be used as carriers of OVs, and which can not only protect the virus from being cleared by the immune system, but also deliver the virus to tumor lesions and release cytokines, thereby enhancing the anti-tumor immune response [181]. In addition, MSCs can also carry chemotherapy drugs such as doxorubicin, paclitaxel, and gemcitabine, and which can inhibit tumor cell growth when anticancer drug-loaded MSCs are co-cultured with tumor cells and administered locally [133]. MSCs can also be modified into suicide genes that convert non-toxic reagents into toxic antitumor drugs, such as human adipose tissue-derived MSCs expressing the suicide gene cytosine deaminase::uracil phosphoribosyltransferase (CD::UPRT) can convert relatively non-toxic 5-fluorocytosine into highly toxic antitumor 5-FU and significantly inhibit tumor growth [182]. In short, based on the tumor-homing characteristics of MSCs, MSCs can be used as a carrier of cytokines, OVs and chemotherapy drugs to target tumors and improve anti-tumor efficacy.

However, the effectiveness of using the tumor homing properties of MSCs to treat tumors will be affected by insufficient homing ability thereby resulting in insufficient targeting and affecting therapeutic efficacy [183]. In order to improve the tumor homing characteristics of MSCs, the P-selectin ligand PSGL-1 mRNA, E-/L-selectin ligand SLeX mRNA, or CXCR4 mRNA were transfected into MSCs, resulting in the rolling and adhesion of the modified MSCs in vascular endothelial cells was enhanced, thereby increasing the homing of MSCs [144]. In addition, overexpression of chemokine receptors (CCR2, CCR3, and CCR4) can enhance the homing ability of MSCs to targets by increasing the migration of MSCs to chemokines [184]. Not only that, loading iron oxide nanoparticles into MSCs can improve the homing ability of MSCs to targets through magnetic guidance [185]. In conclusion, improving the homing ability of MSCs can be achieved through a variety of strategies based on the homing of MSCs involves multiple mechanisms and steps [144].

In addition to using the homing ability of MSCs as a carrier for the treatment of tumors, MSCs also have important value in stem cell transplantation therapy, such as helpful hematopoietic reconstitution after hematopoietic stem cell transplantation, especially for the treatment of leukemia, multiple myeloma and lymphoma [186]. In addition, the immunosuppressive function of bone marrow MSCs can also be utilized to alleviate the possibility of graft versus host disease (GVHD) caused by allogeneic transplantation [187]. Furthermore, allogeneic bone marrow MSCs transplantation rarely causes rejection due to the bone marrow MSCs express low levels of major histocompatibility complex (MHC) class I, MHCII class I, and co-stimulatory molecules (CD40, CD80, and CD86) [188, 189]. Therefore, therapies based on MSCs are a promising way to support hematopoietic stem cell or bone marrow transplantation. In summary, MSCs can not only be used as vectors to target tumors thereby improving anti-tumor efficacy, but also contribute to hematopoietic reconstruction and alleviate GVHD after hematopoietic stem cell transplantation.

A large number of clinical trials have also been carried out on the application of MSCs in tumors. So far, 39 trials have been registered in the ClinicalTrials database, of which 18 trials are aimed at using MSCs to treat cancer (including glioma, myelodysplastic syndrome (MDS), ovarian cancer, head and neck cancer, lung cancer, etc.), 11 trials aimed to use MSCs to treat side effects caused by anti-tumor treatment (including bone marrow suppression, acute kidney injury, cardiomyopathy, erectile dysfunction after rectal cancer surgery, and xerostomia caused by radiotherapy), another 10 clinical trials related to MSCs and hematopoietic stem cell transplantation (5 aimed to investigate the prevention of GVHD after hematopoietic stem cell transplantation by MSCs, and 4 aimed to observe the feasibility or effectiveness of co-infusion of hematopoietic stem cells and MSCs) (Table 1). 12 out of 39 clinical trials on MSCs and tumors have been completed (MSCs for cancer treatment: 4 items, MSCs for reducing side effects of anti-tumor treatment: 2 items, MSCs and hematopoietic stem cell transplantation: 6 items). We have seen the potential of using MSCs therapy in tumors in the published experimental results, such as in the clinical trial “Phase 1 Trial of Celyvir in Children and Adults With Metastatic and Refractory Solid Tumors.” (NCT01844661), where MSCs are used to transport oncolytic adenoviruses (OAds) to the tumor site (referred to as Celyvir therapy). The results showed that OAd MSCs treatment could significantly reduce the tumor growth of osteosarcoma in vivo, and the infiltration of immune cells (especially tumor-infiltrating lymphocytes) in the tumor was higher after treatment. Another clinical trial with published results is “A Phase I/II Trial in Treating Patients With Graft Versus Host Disease by the Infusion of Expanded in Vitro Allogenic Mesenchymal Stem Cell” (NCT00447460), which evaluated the feasibility and efficacy of MSCs infusion with human serum augmentation in the treatment of refractory acute and chronic graft-versus-host disease. The results showed that among the 10 patients treated, 3 patients achieved complete remission, 6 patients achieved partial remission, while only 3 patients did not respond to MSC infusion [190]. The above clinical trial results indicate that the use of MSCs by tumor patients can bring benefits to patients and has broad application prospects.

**Table. 1:** Clinical trials of mesenchymal stem cells and tumors.

Clinical trial purposes	ClinicalTrials.gov Identifier	Conditions	Interventions	Phase	Status
Treatment of tumors	NCT03896568	Recurrent high-grade Glioma	BM-hMSCs-DNX2401	Phase I	Recruiting
	NCT05699811	Locally advanced or metastatic solid tumors	MSC-IFNα	Phase I/II	Recruiting
	NCT01129739	MDS	UC-MSCs/Placenta-derived MSCs	Phase II	Unknown status
	NCT04758533	DIPG/Medulloblastoma	AloCELYVIR (MSCs + ICOVIR-5)	Phase I/II	Recruiting
	NCT05113342	Relapsed/Refractory multiple Myeloma	Allogeneic MSCs (Descartes-25)	Phase I/II	Recruiting
	NCT05047276	Metastatic uveal melanoma	AloCelyvir (MSCs + OVs)	Phase I/II	Not yet recruiting
	NCT05789394	Recurrent GBM	Allogeneic adipose-derived MSCs	Phase I	Recruiting
	NCT02068794	Gynecologic tumors	Adipose tissue derived MSCs	Phase I/II	Recruiting
	NCT02079324	Head and neck cancer	Genetically modified MSCs	Phase I	Unknown status
	NCT01844661	Solid tumors	CELYVIR (bone marrow-derived autologous MSCs infected with ICOVIR5)	Phase I/II	Completed
	NCT04087889	Pancreatic cancer	Allogeneic Adipose-derived MSCs	Not described	Unknown status
	NCT04657315	GBM	MSC11FCD	Phase I/II	Completed
	NCT03298763	NSCLC	MSCTRAIL (TRAIL + MSCs)	Phase I/II	Recruiting
	NCT00851162	Bone neoplasms	MSCs	Phase II/III	Withdrawn
	NCT03184935	MDS	Human UC-MSCs	Phase I/II	Suspended
	NCT02530047	Ovarian cancer	MSC-INFβ	Phase I	Completed
	NCT02804945	Malignancies	MSCs	Phase I	Completed
	NCT01983709	Prostate cancer	Allogeneic human MSCs	Phase I	Terminated
About hematopoietic stem cell transplantation	NCT01092026	Allogeneic stem cell transplantation	MSCs	Phase I/II	Unknown status
	NCT01624701	Chronic leukemia/MDS/ Lymphoma/Myeloma	MSCs	Phase I/II	Terminated
	NCT02032446	Hematologic malignancies	UC-MSCs	Phase I/II	Unknown status
	NCT00361049	cancer	MSCs infusion	Phase I	Completed
	NCT02181478	ALL/AML/NHL/HL/CLL/CML/MDS/MF	MSCs transplantation	Phase I	Completed
	NCT00081055	Hematologic malignancies	Autologous expanded MSCs OTI-010	Phase II	Withdrawn
	NCT00498316	MDS/Leukemia	Cord blood expansion on MSCs	Phase I	Completed
	NCT01045382	Hematologic malignancies	MSCs	Phase II	Terminated
	NCT00504803.	Hematologic malignancies	MSCs infusion	Phase II	Completed
	NCT03106662	Hematopoietic stem cell transplantation	MSCs	Phase III	Completed
	NCT00447460	Patients with hematologic malignancies have GVHD after transplantation	MSCs	Phase I/II	Completed
Reduce the side effects caused by anti-tumor	NCT02648386	Erectile dysfunction after rectal cancer treatment.	NeuroRegen scaffold/UC-MSCs transplantation	Phase I/II	Unknown status
	NCT02509156	Cardiomyopathy due to anthracyclines	Allo-MSCs	Phase I	Completed
	NCT01275612	Cisplatin-induced acute renal failure	MSCs infusion	Phase I	Withdrawn
	NCT02962661	Anthracycline-associated cardiomyopathy	Intravenous infusion or transendocardial injection of MSCs	Phase I	Recruiting
	NCT05672420	Treatment-induced myelosuppression in patients with hematologic malignancies	UC-MSCs	Phase I/II	Not yet recruiting
	NCT04007081	Radiation xerostomia	Autotransplantation of marrow MSCs	Not Applicable	Completed
	NCT04489732 :	Xerostomia following radiotherapy	MSCs	Phase I	Active, not recruiting
	NCT03874572	Radiation-induced hyposalivation and xerostomia	Allogeneic MSCs	Phase I	Active, not recruiting
	NCT05820711	Patients with xerostomia after radiotherapy for head and neck cancer	MSCs	Phase I	Recruiting
	NCT04776538	Xerostomia following radiotherapy	MSCs	Phase II	Recruiting
	NCT03876197	Xerostomia due to radiotherapy	Autologous adipose-derived MSCs	Phase I/II	Enrolling by invitation

*GBM* glioblastoma, *DIPG* diffuse intrinsic pontine glioma, *NSCLC* non-small cell lung cancer, *ALL* acute lymphoblastic leukemia, *AML* acute myelogenous leukemia, *NHL* non-Hodgkin lymphoma, *HL* Hodgkin lymphoma, *CLL* chronic lymphocytic leukemia, *CML* chronic myelogenous leukemia, *MDS* myelodysplastic syndromes, *MF* myelofibrosis, OVs oncolytic viruses. *UC-MSCs* umbilical-cord-derived MSCs, *allo-MSCs* allogeneic human mesenchymal stem cells, *TRAIL* tumor necrosis factor-associated apoptosis-inducing ligands, *GVHD* Graft versus host disease.

MSCs as another large group of cells in TASCs which have tumor homing ability and play different roles at different stages of tumors, such as exogenous MSCs have the effect of inhibiting tumorigenesis and growth, but MSCs homing to tumor sites can promote tumor occurrence and development through a variety of mechanisms. Moreover, due to the ability of MSCs to spontaneously invade tumors guided by chemokines in TME, MSCs can be used as a carrier to target tumors to exert anti-tumor effects through delivering anti-cancer cell factors, pro-apoptotic proteins, suicide genes, OVs and chemotherapy drugs. In addition, MSCs can also help hematopoietic reconstruction, alleviate GVHD, and alleviate the side effects of anti-tumor therapy after hematopoietic stem cell transplantation, so using some of the properties of mesenchymal stem cells to treat tumors is a promising strategy.

### Cancer-associated adipocytes

Adipocytes are considered to be an inert cell population, however, adipocytes located in TME are “activated” by tumor cells with a pro-tumor phenotype, and these adipocytes are called “tumor-associated adipocytes (CAAs)”. CAAs are mainly derived from MSCs or undifferentiated adipocyte precursors in adipose tissue matrix, and a small number of CAAs can also come from CSCs [191]. CAAs can promote tumor occurrence and development through the secretion of adipokines, inflammatory factors, and the production of fatty acids [192]. It is possible to target CAAs for tumor treatment based on the tumor-promoting effect of CAAs. We will further introduce the relationship between CAAs and tumors, the mechanism of CAAs promoting tumors, and the strategies of targeting CAAs for the treatment of tumors.

### Cancer-associated adipocytes and tumors

CAAs are an important component of cellular composition in TME, especially in tumors such as breast cancer, ovarian cancer, prostate cancer, kidney cancer, gastric cancer, and colon cancer [193]. The crosstalk between adipocytes and cancer cells can lead to changes in the phenotype and function of adipocytes, such as tumor-derived soluble factors TNF-α, IL-6, plasminogen activator inhibitors 1, Wnt3a, and exosomes microRNAs (such as miR-144, miR-126, and miR-155) can act on adipocytes at the forefront of tumor invasion and further induce the formation of CAAs [2]. Moreover, activated CAAs differ from normal adipocytes in morphology and function, they exhibit decreased adipocytes/macrophage fatty acid-binding protein 2 (Ap2) and fatty acid-binding protein 4 (FABP 4), while the expression of MMP11 and the release of IL-6, IL-1β, IGFBP-2 increased [194, 195].

Cancer cells are more likely to recruit adipocytes compared with normal tissues, and the adipocytes recruited into TME participate in tumorigenesis, promoting tumor growth and invasion [196, 197]. For example, the accumulation of adipose tissue adjacent to tumor tissue is related to the increase of incidence rate, progress, and metastasis of breast cancer [198]. Mature adipocytes in vitro can significantly promote the proliferation of breast cancer cells (MCF-7) and normal breast cells (184B5) [199]. At the same time, co-culture of mature adipocytes and breast cancer cells can promote cancer cell growth [200] and mediate EMT by enhancing the expression of MCF7 cell Forkhead Box C2 (FOXC2), twist family bHLH transcription factor 1 (TWIST1), and N- and E-cadherin [201]. In addition, adipocytes co-cultured with breast cancer cells can promote the invasion of tumor cells by overexpressing the inflammatory cytokines such as IL-6, TNFα, and MMPs [202]. Moreover, the production of IL-8 and fatty acid-binding protein 4 by adipocytes increased after co-culture of human adipocytes and ovarian cancer cells thereby promoting the homing, migration, and invasion of cancer cells [203]. In conclusion, adipocytes play an important role in the occurrence, development and metastasis of cancer, especially in breast cancer with rich adipose tissue [204]. At the same time, adipocytes also promote tumor cell resistance to anti-tumor treatment, such as adipose stromal cells inhibit the tolerance of prostate cancer cells to docetaxel, cabazitaxel and CDDP [205]. The exosome microsomal triglyceride transfer protein (MTTP) derived from adipocyte can inhibit iron ptosis in colorectal cancer and promote tumor resistance to chemotherapy [206].

### Protumor mechanisms of cancer-associated adipocytes

**Fig. 4:**
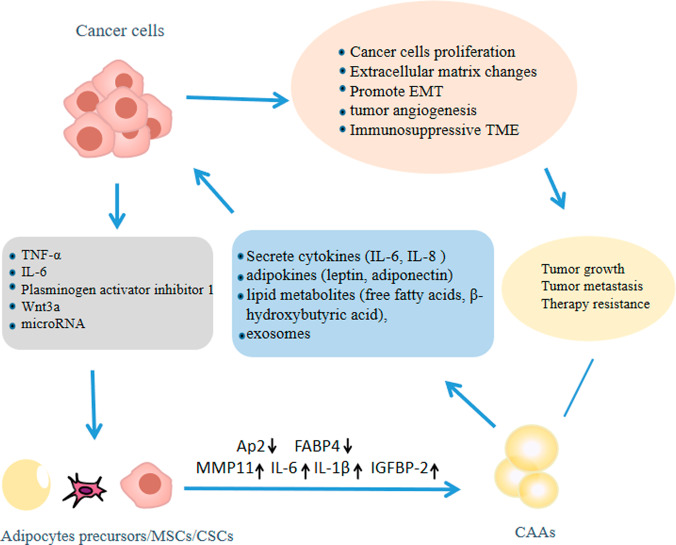
Complex relationship between tumors and cancer-associated adipocytes (CAAs): tumors induce the formation of CAAs by adipocytes/mesenchymal stem cells (MSCs)/cancer stem cells (CSCs) by secreting soluble factors tumor necrosis factor-α (TNF-α), Interleukin-6 (IL-6), plasminogen activator inhibitor 1, Wnt3a, and exosomes. Activated CAAs showed a decrease in adipocyte markers such as adipocytes/macrophage fatty acid-binding protein 2 (Ap2) and fatty acid-binding protein 4 (FABP4), while the expression of MMP11 and the release of IL-6, IL-1β, and IGFBP-2 increased. CAAs secrete cytokines, adipokines, lipid metabolites, and exosomes to promote the proliferation of cancer cells, regulate extracellular matrix (ECM) structure, form an immunosuppressive microenvironment, promote tumor angiogenesis and epithelial interstitial transformation (EMT), and ultimately promote tumor growth, metastasis and resistance to anti-tumor treatment.

CAAs can promote tumorigenesis and development by secreting a large number of cytokines (e.g., IL-6, IL-8, and chemokines), adipokines (leptin, adiponectin, autotaxin, and resistin), lipid metabolites (free fatty acids and β-hydroxybutyric acid), and exosomes [192, 207] (Fig. 4). Among them, cytokines derived from adipocyte, such as leptin, globular adiponectin, resistin, IGFBP-2, and CCL5, can be used as paracrine signals of cancer cells to upregulate invasion related proteins such as calcium binding protein S100A7, MMP-9, and urokinase type plasmin activator (UPA), thereby promoting the invasion and migration of cancer cells [208–210]. In addition, CAAs-induced TGF-β, secreted IL-8, and produced leptin can mediate EMT in cancer cells, promote tumor spread and tumor angiogenesis, respectively [211–214].

CAAs can also promote tumor cell invasion by upregulating the levels of versican and leptin in renal cancer cell lines [215]. In addition, the high expression of epidermal FABP5 in adipocytes can also lead to the development and metastasis of cancer [216]. CAAs can also support tumor progression by secreting lipid metabolites in addition to secreting cytokines and expressing invasion proteins to promote tumor development and metastasis. For example, cancer cells stimulate the breakdown of fat into fatty acids (FA) in CAAs, and FA enters cancer cells through specific fatty acid receptors and binding proteins (such as CD36 and Fatty Acid Transport Protein 1) for membrane synthesis and energy metabolism (β-oxidation), or lipid-derived cell signaling (derivatives of arachidonic acid and linolenic acid) [197]. Among them, CAAs release essential fatty acids (FFA) that are absorbed by cancer cells to promote growth and proliferation [217, 218]. The released free FA can also activate and regulate other cells such as macrophages, vascular endothelial cells and muscle cells, which is conducive to the formation of the original cancer microenvironment, and FABP4 released by CAAs is an energy source carrier for cell invasion [219]. In summary, CAAs can participate in the occurrence and development of tumors through mechanisms such as the secretion of cytokines, lipid metabolites, and expression of invasion proteins.

In addition, adipocytes can also support tumor progression by regulating immune cells in TME, such as the levels of exosome microRNA (miRNA)-155 increased during co-culture of tumor cells and adipocytes, thereby recruiting macrophages and promoting their differentiation into TAMs that support tumor development [220–222] In addition, visfatin secreted by CAAs can also induce M2 macrophage polarization and accelerate the glycolysis process in malignant tumor cells [223]. Not only that adipocytes can also acquire myofibroblast and macrophage-like features through metabolic reprogramming and dedifferentiation (that is “adipocytes-stromal switch”) thereby promoting tumor progression [224]. Adipocytes co-cultured with gastric cancer cells can also be converted into CAFs to promote tumor progression and peritoneal metastasis [225]. In addition, CAAs can also differentiate into PCs and participate in the formation of blood vessel walls [226]. Moreover, the interaction between various lipids, cytokines, and adipokines secreted by bone marrow adipocytes and cancer cells can promote bone metastasis in solid tumors, especially prostate cancer, breast cancer, lung cancer, and multiple myeloma [227]. In summary, CAAs can participate in the progression of tumors through various mechanisms.

The tumor-promoting mechanism of CAAs can be realized by multiple signaling pathways, such as IL-6 and leptin derived from CAAs can promote tumor metastasis by activating the JAK/STAT3 and PI3K/AKT signaling pathways to promote the expression of lysine hydroxylase (PLOD2) [228] And the cytokines LIF secreted by CAAs can promote the migration and invasion of breast cancer cells through the STAT3 signaling pathway [229]. In addition, the elevated exosome miRNA-155 during the co-culture of tumor cells and adipocytes can promote the production and release of adipocytes CCL2 and CCL5 by targeting the SOCS6/STAT3 pathway, thereby regulating the function and polarity of macrophages and promoting tumor progression [221]. In summary, CAAs can participate in tumor progression and metastasis by secreting cytokines, adipokines, providing metabolites, and transdifferentiation into other cells, and the tumor-promoting mechanism of CAAs can be realized through multiple signaling pathways.

### Strategies for targeting cancer-associated adipocytes to treat tumors

Transforming CAAs into normal adipocytes, inhibiting related bioactive molecules, and exosomes are effective methods for targeting CAAs in the treatment of tumors. For example, metformin can exert anti-tumor effects by regulating adipocyte leptin and reversing dysfunctional adipocytes and normalizing them [230]. In recent years, it has also been found that metformin has a significant inhibitory effect on the growth and adipocyte differentiation of human adipose stromal cells (ADSCs) [231]. In addition, treatment targeting bioactive molecules secreted by CAAs can also inhibit tumors, such as Peroxisome proliferator-activated receptor-γ (PPAR-γ) agonists can inhibit tumors by reversing disorders of bioactive molecules such as upregulation of adiponectin, and PPAR-γ agonists such as rosiglitazone and pioglitazone can also promote adipocyte differentiation by downregulating factors such as leptin, IL-6, and TNF-α [232, 233]. Moreover, peptide analogues located at the binding site of leptin and leptin receptor (ObR) can selectively inhibit the interaction between leptin and leptin receptor, thereby inhibiting tumor occurrence and metastasis [234]. In addition, inhibiting the exosomes miRNA-155 of tumor cells can reduce the levels of CCL2 and CCL5 in CAAs co-culture thereby inhibiting tumor growth [221]. At the same time, blocking fatty acid-derived lipid uptake or lipid-related metabolic pathways in cancer cells is also an effective therapeutic strategy for lipid-rich cancers [197]. In summary, targeted CAAs for tumor therapy can be achieved through a variety of strategies.

As an important component of the tumor matrix, CAAs can promote the survival, proliferation, and migration of tumor cells through mechanisms such as secretion of tumor-related adipocytokines, inflammatory factors, and production of fatty acids. They can also work with other stromal cells to promote tumor progression. In addition, the interaction between adipocytes and tumor cells can also increase resistance to anti-tumor therapy. In summary, the activated CAAs in TME promote tumor progression, so strategies such as converting CAAs into normal adipocytes, inhibiting related bioactive molecules, and exosomes are an option for treating tumors based on CAAs.

### Tumor-associated endothelial cells

The formation of blood vessels in tumors is essential for tumor growth and metastasis, and tumor blood vessels not only provide oxygen and nutrients for tumors to support tumor growth but also provide a channel for tumor metastasis [235]. Among them, vascular endothelial cells are important tissues for maintaining hemoperfusion, and TECs interact with tumor cells to form neovascularization thereby supporting tumor development, promoting tumor metastasis and participating in the resistance of anti-tumor therapy [236]. Therefore, it is possible to target vascular endothelial cells to treat tumors based on these effects of TECs on tumors, such as macromolecular anti-angiogenic drugs (AADs) represented by bevacizumab have been widely used in clinical practice. In addition, a variety of small-molecule kinase inhibitors have also seen significant efficacy. Next, we will introduce endothelial cells in tumors in detail.

### Tumor blood vessels and tumor-associated endothelial cells

Angiogenesis is strictly regulated by the balance of angiogenic factors and antiangiogenic factors [237], but there is an imbalance between these two factors in tumors, with levels of angiogenic activators higher than those of angiogenic inhibitors [238]. For example, VEGF upregulateion due to hypoxia, activation of oncogenes and mutation of tumor suppressor genes in tumors, which activates ECs through paracrine signals to stimulate ECs proliferation, induce angiogenesis and enhance vascular permeability [239]. In addition to VEGF, cancer cells also secrete other angiogenic factors such as basic FGF, angiopoietin (Ang), HGF, epidermal growth factor, PDGF, and placental-derived growth factor [239], and all of which can significantly increase the proliferation, migration, and vascular formation of ECs [236, 238]. These overexpressed proangiogenic factors can induce the transition of ECs from a quiescent state to an active state, thereby enabling them to acquire a more migratory and invasive phenotype [240]. At the same time, the expression of angiogenesis inhibitory genes are downregulated in TME such as thrombospondin-1 (TSP-1) [241]. In conclusion, angiogenic factors are elevated in tumors and angiogenesis inhibitory genes are downregulated, and elevated angiogenic factors can induce tumor vascularization by promoting the proliferation and migration of ECs.

Tumor blood vessels are different from normal blood vessels in phenotype and morphology, and tumor blood vessels are chaotic, unhierarchical, abnormally dilated, and have the characteristics of high penetration and low perfusion. In addition, these vessels do not serve as normal barriers due to the lack of proper perivascular coverage and tight EC connections [242–244]. Not only that, the TECs that make up tumor blood vessels are also significantly different from normal endothelial cells (NECs), which are fragile, leaky, and highly proliferative and angiogenic [245]. Moreover, TECs are irregular in shape and size [237], and cells proliferate and migrate faster [229]. At the same time, TECs also manifest as cytogenetic abnormalities, such as both TECs and circulating TECs show chromosome instability characteristics such as aneuploidy, chromosome translocation and chromosome deletion [243]. Furthermore, TECs are also different from NECs at the molecular level, for example, TECs express embryonic markers such as renal transcription factor Paired Box2 (PAX2) [246] and CSCs markers such as stem cell antigen 1 (SCA-1) and CD90, and upregulate multidrug resistance (MDR) 1, Aldehyde dehydrogenase (ALDH), VEGF and VEGFR2 [247]. In addition, TECs also have metabolic plasticity and reprogramming. For example, TECs have increased glucose uptake and higher glycolysis rate than NECs [248, 249]. Not only that, fatty acid (FA) and serine biosynthesis pathways have also undergone changes [243], such as upregulation of FASN, PHGDH, and phosphoserine aminotransferase 1 (PSAT1) [238, 250]. Moreover, the phenotype of TECs also changes with tumor progression, such as TECs isolated from metastatic tumors have more chromosomal abnormalities, higher proliferation index and invasion potential, expression of more vascular secretory factors, more ability to attract and adhere to tumor cells, and resistance to anticancer drugs (5-FU, paclitaxel) than TECs isolated from non-metastatic tumors [238, 243]. In summary, tumor blood vessels differ from normal blood vessels in phenotype and morphology, and the TECs that make up tumor blood vessels exhibit significant differences from NECs in morphology, chromosome, molecular level, and metabolism.

**Fig. 5:**
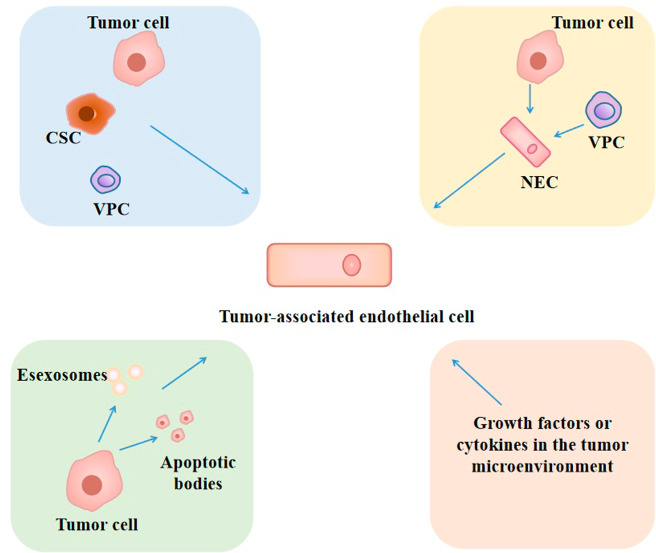
Possible mechanisms of tumor endothelial cells (TECs) abnormalities. TECs can be formed by transdifferentiation of tumor cells, cancer stem cells (CSCs), or vascular progenitor cells (VPCs), and TECs can also be formed by fusion of normal endothelial cells (NECs) with malignant tumor cells or VPCs. TECs can also absorb apoptotic bodies or exosomes released by tumor cells, which can absorb tumor oncogenes. In addition, growth factors or cytokines in the tumor microenvironment can lead to genetic instability of TECs, thereby leading to TECs abnormalities.

The mechanisms that cause TECs to be abnormal may be related to the source of TECs (Fig. 5). For example, TECs can be transdifferentiated and formed by inducing tumor cells, CSCs, or vascular progenitor cells (VPCs) through mechanisms such as autophagy, and ROS activation of Akt/inhibitor of kappa B kinase (IKK) signaling pathways, such as CSCs of glioblastoma, breast, and ovaries all can differentiate into ECs in morphology and function [242, 243, 251, 252]. In addition, TECs can also be formed by the fusion of NECs with malignant tumor cells. ECs can also take up apoptotic bodies or exosomes released by tumor cells thereby taking up tumor oncogenes. In addition, the mechanism that causes TECs to be abnormal is also related to the genetic instability of TECs due to the influence of growth factors or cytokines in TME [243]. In summary, a variety of mechanisms lead to TECs being different from NECs thus resulting in TECs having different properties from NECs.

### Tumor-associated endothelial cells and tumors

TECs can participate in the occurrence, development, and metastasis of tumors by secreting cytokines such as interleukin, VEGF-A, and Heat Shock 70 kDa Protein 12B HSPA12B, as well as exosomes to activate receptors on tumor cells, support tumor metabolism, or suppress anti-tumor immune responses [236, 253–257]. In addition, TECs can promote cancer cell progression and metastasis by expressing endothelial protein C receptor (EPCR) and NOTCH1 [7, 258]. At the same time, TECs can also recruit macrophages and induce them to differentiate into M2 TAM, thereby inhibiting anti-tumor immune responses [257]. Moreover, TECs can also use MMPs to destroy the basement membrane and cause pericyte separation, endothelial cell migration, and degradation of extracellular matrix, thus contributing to tumor growth and metastasis [243]. In summary, TECs can participate in the occurrence, development, and metastasis of tumors through various mechanisms.

In addition, TECs are also involved in tumor resistance to antitumor treatments, such as kidney cancer-derived TECs resistant to vincristine [259], and liver cancer-derived TECs resistant to 5-FU and doxorubicin [260]. TECs can also resist paclitaxel therapy by upregulating P-glycoprotein (P-gp, ABCB1) [261]. The mechanism of TECs leading to tumor drug resistance may be related to the cytogenetic abnormalities of TECs leading to genetic instability [243]. In addition, exosomes secreted by cancer cells can also induce TECs to resist antitumor drugs, such as miRNA-1246 secreted by cancer cells can induce TECs to be resistant to 5-FU [262]. In summary, TECs are not only involved in the progression of tumors but also in the resistance of tumors to anti-tumor therapy.

### Therapeutic strategies targeting tumor-associated endothelial cells

The strategy of anti-angiogenesis therapy for tumors is already relatively mature in clinical practice. Up to now, hundreds of AADs have been developed, among which TECs are the main targets of anti-angiogenic therapy. AADs can be divided into three categories according to the mechanism of action of AADs: anti-angiogenesis monoclonal antibody (AA-MA), anti-angiogenesis tyrosine kinase inhibitor (AA-TKI) and endogenous angiogenesis inhibitor. Among them, AA-MA mainly binds to VEGFA or vascular endothelial growth factor receptor (VEGFR) 2, while AA-TKI mainly inhibits targets such as VEGFR, PDGFR, and c-Kit. Endogenous angiogenesis inhibitors mainly exert anti-angiogenic effects by downregulating the expression of VEGF and its receptors. Currently, more than 10 anti-angiogenic drugs are approved by the FDA or NMPA, including AA-MA: Bevacizumab, Aflibercept, and Ramucirumab. AA-TKI: Sorafenib, Sunitinib, Pazopanib, etc, and endogenous angiogenesis inhibitors: Endostar. These drugs all can exert antitumor effects by acting on TECs [236]. For example, Bevacizumab inhibits endothelial mitosis, Aflibercept reduces vascular endothelial permeability [263], Endostar induces endothelial apoptosis [264], Sorafenib inhibits endothelial cell proliferation [265], etc. Table 2 lists the current AADs approved by the Food and Drug Administration (FDA) and the National Medical Products Administration (NMPA) for the treatment of cancer.

**Table. 2:** FDA- and NMPA-approved AADs for cancer treatment.

Classification	Name of the drug	The target of action	Approved tumors for treatment	Approved time and institution
Anti-angiogenic monoclonal antibodies	Bevacizumab	VEGFA	NSCLC/mCRC/mRCC/OVC/CC/BC/GBM	2004 (FDA)
	Aflibercept	VEGFA/B, PIGF	mCRC	2012 (FDA)
	Ramucirumab	VEGFR2	NSCLC/HCC/mCRC/GC/GEJA	2005 (FDA)
Endogenous angiogenic inhibitors	Recombinant human endostatin injection	VEGF, HIF-1α, MMPs, bFGF	NSCLC	2006 (NMPA)
Antiangiogenic tyrosine kinase inhibitors	Sorafenib	VEGFR2/3, PDGFR-β, c-Kit, FLT-3, et al.	HCC/RCC/TC	2005 (FDA)
	Sunitinib	VEGFR1/2/3, PDGFRα/β, Kit, FLT-3, RET, et al.	GIST/PAAD/mRCC	2006 (FDA)
	Pazopanib	VEGFR1/2/3, PDGFRβ, FGFR1, c-Kit.	RCC/STS	2009 (FDA)
	Vandetanib	VEGFR2, EGFR, RET.	Medullary TC	2011 (FDA)
	Axitinib	VEGFR1/2/3, PDGFRβ, c-Kit.	RCC	2012 (FDA)
	Regorafenib	VEGFR1/2/3, PDGFRα/β, KIT, et al.	mCRC/HCC/GIST	2012 (FDA)
	Ponatinib	Abl, PDGFRα, VEGFR2, FGFR1.	AML/CML	2012 (FDA)
	Nintedanib	VEGFR1/2/3, FGFR1/2/3, PDGFRα/β.	NSCLC	2014 (FDA)
	Apatinib	VEGFR2	GC/GEJA/HCC	2014 (NMPA)
	Lenvatinib	VEGFR1/2/3, PDGFRα/β, FGFR1/2/3/4, FGFR1-4, Kit, RET.	HCC/EC/RCC/TC	2015 (FDA)
	Cabozantinib	VEGFR1/2/3, MET, RET, Kit, Axl, et al.	differentiated TC/medullary TC/HCC/RCC	2016 (FDA)
	Anlotinib	VEGFR, PDGFR, FGFR, c-Kit	NSCLC/STS	2018 (NMPA)
	Fruquintinib	VEGFR1/2/3	mCRC	2018 (NMPA)
	Erdafitinib	FGFR2/3/4, PDGFRα/β, VEGFR2, RET, FLT4, Kit, et al.	UC	2019 (FDA)
	Tivozanib	VEGFR1/2/3, PDGFRβ, c-Kit	RCC	2021 FDA

*NSCLC* non-small cell lung cancer, *mCRC* metastatic colorectal cancer, *mRCC* metastatic renal cell carcinoma, *OVC* ovarian epithelial cancer, *CC* cervical cancer, *BC* breast cancer, *GBM* glioblastoma, *HCC* hepatocellular carcinoma, *GC* Gastric adenocarcinoma, *GEJA* gastroesophageal junction adenocarcinoma, *TC* thyroid cancer, *GIST* gastrointestinal stromal tumor, *PAAD* pancreatic cancer, *EC* endometrial carcinoma, *UC* urothelial carcinoma, *STS* soft tissue sarcoma, *AML* acute lymphoblastic leukemia, *CML* chronic myelogenous leukemia.

In addition to acting on VEGF/VEGFR affecting ECs in the treatment of tumors, peroxisome proliferator-activated receptors (PPARs) and vitamin D receptors (VDR) are also targets for the treatment of tumors targeting ECs, such as PPARα ligands have a powerful effect in inhibiting endothelial cell proliferation and angiogenesis, and the PPARα agonist fenofibrate can inhibit endothelial cell proliferation and VEGF production thereby inhibiting tumor growth [266]. In addition, calcitriol, which acts on VDR, also has an inhibitory proliferative effect on TECs but has no inhibitory effect on normal ECs [267]. Not only that, treatment targeting 17β-estradiol can also treat tumors by affecting ECs since 17β-estradiol can increase tumor vascular density and stabilize the vascular endothelial system [268].

In addition to acting on targets such as VEGF, PPARs, VDR, and 17β-estradiol to affect ECs to treat tumors, silencing-related genes can also treat tumors through ECs. For example, ablation of the A Disintegrin And Metalloprotease (ADAM) 17 gene in ECs and drugs that inhibit ADAM17 can prevent long-term metastases formation in the lung [269]. At the same time, knockout of Homologous to the E6-associated protein carboxyl terminus domain containing 3 (HECTD3) in ECs can also significantly inhibit lung colonization of tumor cells [270]. In addition, based on the fact that Notch signaling is essential for vascular development and tumor angiogenesis, inhibiting Notch ligand Delta like 4 or inhibiting Notch signaling in endothelial cells can lead to endothelial dysfunction, disrupt new angiogenesis, and limit tumor growth [271, 272].

Moreover, ECs L-type amino acid transporter 1 (LAT1)-mediated amino acid transport is the basis for supporting ECs proliferation and in vitro translation initiation, so therapeutic inhibition against LAT1 can also inhibit angiogenesis [273]. In addition, tumor progression can also be inhibited by regulating the metabolism of ECs. For example, inhibition of COX-2 in ECs can induce normalization of glucose metabolism thereby inhibiting tumor progression [274]. At the same time, the fatty acid synthase (FASN) blocker orlistat can also inhibit ECs proliferation, and knocking out FASN in endothelial cells can prevent vascular sprouting by reducing ECs proliferation [275]. In short, ECs can be inhibited by targeting receptors, silencing genes, inhibiting signaling pathways, and regulating ECs metabolism, thereby inhibiting their tumor-promoting effects.

TECs are another type of stromal cells in TME, in which tumor cells interact with TECs to form tumor blood vessels that are different from normal blood vessels in phenotype and morphology, and ultimately promote tumor development and metastasis. Not only that, TECs are also involved in drug resistance to anti-tumor treatment. Based on the influence of TECs on tumors and anti-tumor therapy, the treatment targeting vascular endothelial cells has become an effective anti-cancer treatment. For example, most of the current anti-angiogenic therapies target endothelial cells, such as large molecule antibodies and small molecule kinase inhibitors that are widely used in clinical practice. In addition, it can also inhibit ECs thereby inhibiting tumors by targeting other targets such as PPARs, VDR, 17β-estradiol, etc, as well as silencing genes and inhibiting signaling pathways. In summary, weakening the complex relationship between ECs and cancer cells, and finding therapeutic strategies that can target TECs with little interference with the normal vascular system is the direction of targeting ECs to treat tumors.

### Tumor-associated pericytes

Pericytes (PCs) also known as parietal cells which are located on the inner surface of blood vessels and interact with newly proliferated ECs to play an important role in regulating vascular stability, permeability and maintaining the integrity of the blood-brain barrier. In addition, PCs are directly involved in the supply of oxygen, nutrient, and removal of waste [276]. In terms of tumor promoting mechanisms, PCs not only regulate angiogenesis and remodeling but also participate in tumor progression through various mechanisms, such as regulating immune responses in TME, secreting soluble factors, and transforming into other stromal cells [276]. We will introduce PCs in TME below.

### Pericytes and tumors

PCs do not have specific surface markers and mainly express PDGFRβ, CD146 (also known as the melanoma cell adhesion molecule MCAM), Neuron-glial antigen 2 (NG2), CD13 (aminopeptidase N), and α-SMA, but these markers are also expressed on other types of cells such as endothelial cells and smooth muscle cells [277]. During tumorigenesis, PCs can be formed by TGF-β-induced epithelial cell transformation or by transdifferentiation of tumor cells or activated fibroblasts in TME [278, 279]. PCs themselves are also highly plastic, they can differentiate into different cell populations such as fibroblasts, fat cells, myoblasts, smooth muscle cells, and bone and chondrocytes [280]. For example, PCs expressing chondroitin sulfate proteoglycans (NG2 proteoglycans) may be the origin cells of stromal tumors such as bone and soft tissue sarcomas [281].

During angiogenesis, ECs can recruit PCs into new blood vessels through activating PDGFR signal by secreting PDGF β [282]. PCs in tumors are important mediators for maintaining the integrity of tumor blood vessels, and they interact with ECs to form a dysfunctional, leaky, and dysfunctional tumor vascular system [276]. Moreover, the coverage of PCs in tumor blood vessels is relatively low, which leads to tumor cell extravasation, increased plasma volume in the tumor interior/interstitium, and increased local pressure, thereby promoting tumor progression and metastasis [283]. In addition, PCs also play an important role in the formation of niche before cancer metastasis, such as PCs leaving blood vessels under the action of primary tumor factors in lung metastasis, and PCs detach from pulmonary blood vessels after primary tumor implantation into the lung and expand into stromal producing cells in the lung parenchyma before metastasis [284] thereby forming a microenvironment that is easy to be colonized by cancer cells [285]. Furthermore, the PCs in tumors are different from those in normal tissues, the PCs in tumors exhibit abnormalities and functional disorders [286, 287]. In addition, the glycolysis driven by hexokinase 2 (HK2) is increased in tumor PCs, and which up-regulates their ROCK2-MLC2 mediated contractility leading to impaired blood vessel supporting function [288]. In summary, TECs are different from PCs in normal tissues, and TECs can interact with ECs to form dysfunctional tumor blood vessels and participate in tumor progression and metastasis.

### Pro-tumor mechanism and resistance to antitumor therapy of Pericytes

**Fig. 6:**
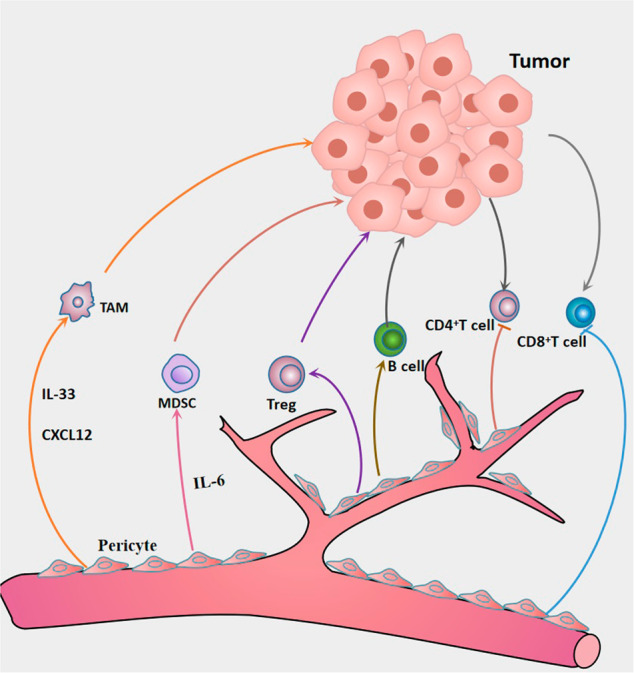
The immunoregulation of pericyte in the tumor microenvironment: Pericytes (PCs) can recruit tumor-associated macrophages (TAMs) by releasing IL-33, CXCL12, etc. PCs can induce the expression of IL-6 thereby increasing the migration of bone marrow-derived suppressor cells (MDSCs). PC can also increase the recruitment of regulatory T cells (Treg) and B cells. Not only that, PCs may be an inhibitor of CD8^+^ T cells infiltration. It is also a negative regulator of CD4^+^ T cells, inducing the incompetence of CD4^+^ T cells. PCs can also inhibit T cells proliferation by releasing PGE2, NO, HGF, etc.

PCs as essential components of TME, which not only work with ECs to promote tumor growth and metastasis, but also promote tumor growth and progression by regulating the immune response in TME, secreting soluble factors, and converting to other stromal cells [276]. For example, PCs can affect the number of immune cells, and studies have shown that the coverage of PCs is related to the migration and infiltration of immune cells [289], and PCs can attract innate white blood cells through microvenous outflow by upregulating the expression of the adhesion molecule Intercellular Cell Adhesion Molecule (ICAM)-1 and releasing the chemokine macrophage migrationinhibitory factor (MIF) [290]. More and more evidence show that PCs in TME have regulatory effects on both innate and adaptive immunity (Fig. 6). For example, PCs can recruit MDSCs, and PCs-derived IL-33, CXCL12, and CXCL14 can promote macrophage recruitment and polarize towards M2 like phenotype [276, 291, 292]. In addition, human malignant glioma-derived pericytes (HMGPs) can inhibit T cell proliferation by releasing Prostaglandin E2 (PGE2), nitrous oxide (NO), serum human leukocyte antigen G (sHLA-G), and HGF, and can enhance the expression of anti-inflammatory cytokines TGF-β and IL-10 by upregulating chaperone-mediated autophagy (CMA), thereby recruiting Tregs and inhibiting the activity of T cells and antigen-presenting cells [293]. CD90-positive PCs in malignant gliomas are inhibitors of leukocyte and CD8^+^ T cells infiltration [293]. Moreover, PCs are also negative regulators of CD4^+^ T cells, which can inhibit the proliferation and activation of CD4^+^ T cells [276]. Furthermore, PCs activated by cancer cells can also inhibit anti-tumor immune responses by downregulating the expression of CD80, CD86 co-stimulatory molecules and MHC-II, as well as upregulating the expression of PD-L1 [294, 295]. In summary, PCs can affect immune cells in TME and inhibit anti-tumor immune responses through various mechanisms.

In addition to their immunomodulatory effects, PCs can also influence tumor progression through mechanisms such as transforming into other stromal cells and secreting cytokines. For example, PCs are considered to be one of the main sources of CAFs, and research has shown that cancer cells can induce the transformation of PCs into CAFs by activating the PI3K/AKT and MEK/ERK pathways, thereby promoting tumor invasion and metastasis [296]. In addition, PCs also have chemical attraction, adhesion, and proliferation-promoting effects on cancer cells by secreting soluble factors such as CCL2, CCL3, CXCL1, IFN- γ, and IL-8 [276, 285]. Among which PC-derived CCL2 can also improve tumor cell survival and tumor growth by stimulating MEK1-ERK1/2-Rho-associated coiled spiral protein kinase 2 (ROCK2)-dependent signaling in tumor cells [286]. Moreover, PCs can also promote local adhesion formation of tumor cells by secreting ECM, and exhibit migratory and invasive phenotypes by inhibiting intercellular adhesion and E-cadherin expression [297]. PCs can also promote EMT by secreting TGF-β1, which in turn contributes to cancer cell invasion [298]. Not only that PCs also play a crucial role in the occurrence and development of brain metastases, such as peribrain cells have a chemotaxis effect on cancer cells by secreting a large number of extracellular matrix proteins, and can also enhance the adhesion of cancer cells, and promote the proliferation of cancer cells by secreting IGF2 [4]. In conclusion, cytokines secreted by PCs can promote tumor progression and invasion through a variety of mechanisms.

In addition, PCs also mediate the resistance of tumors to anti-tumor therapy. For example, during the process of tumor growth, the proliferation of PCs leads to vascular abnormalities that affect the delivery of anti-tumor drugs. Moreover, PCs can also participate in tumor resistance to treatment by secreting cytokines. For example, PCs in gliomas can promote DNA repair and induce resistance to temozolomide (TMZ) drugs through secreting CCL5 and binding to CCR5 expressed on glioblastoma multiforme (GBM) cells, conversely, silencing the CCL5-CCR5 signal can improve the efficacy of TMZ [299]. In addition, PCs can also protect ECs from the toxicity of chemotherapy and mediate the efficacy of anti-angiogenic drugs and other molecular targeted therapies [300, 301]. For example, PCs can adaptively increase their coverage around tumor vessels (BVs) and induce tumors to resist antiangiogenic therapy by secreting proangiogenic factors or other soluble factors [276]. In summary, PCs can participate in tumor resistance to anti-tumor treatment through multiple mechanisms.

### Therapy with targeted pericytes

PCs can promote tumor growth, metastasis, and resistance to anti-tumor treatment through a variety of mechanisms, so treatments of PCs can also treat tumors. For example, targeting glioma stem cell-derived PCs in gliomas can disrupt the blood tumor barrier (BTB) in GBM thereby leading to increased leakage of chemotherapy drugs and improving drug delivery and anti-tumor efficacy [289]. In addition, inhibiting the glycolytic activator fructose-2,6-diphosphatase 3 (PFKFB3) in ECs not only induces normalization of tumor blood vessels, but also increases the coverage of PCs and the adhesion of tumor blood vessels to ECs, thereby increasing the delivery of chemotherapy drugs [248]. Furthermore, due to the high proportion of hexokinase 2 (HK2)-positive PCs in tumors, reducing HK2/ROCK2 through shRNA or inhibitors can reduce the contractility of tumor PCs to rebuild the vascular system and thus improve drug delivery. For example, the combination of HK2 inhibitor 3-bromopyruvate and doxorubicin therapy can enhance the inhibitory effect of chemotherapy drugs on tumor growth in animal models of lung and liver cancer [302, 303].

In addition to influencing PCs by acting on metabolism-related enzymes, the inactivation of some genes and the inhibition of corresponding signals can also inhibit tumor growth through PCs. For example, inactivation of the Kruppel like factor 4 (KLF4) gene in PCs can reduce colonization (metastasis) of lung tumor cells by reducing the formation of extracellular fibrobinding proteins [284]. In addition, silencing IGF2 signaling or inhibiting IGF2 signaling with matrine (PPP) can block the promoting effect of PCs on breast cancer cell proliferation [304]. At the same time, based on PCs protecting ECs against angiogenic drugs, tyrosine kinase inhibitors which eliminate PDGFRβ-positive PCs combined with VEGF inhibitors are more effective at blocking tumor angiogenesis than anti-VEGF alone [305]. Such as a tyrosine kinase inhibitor imatinib targeted the PDGFRβ receptor upregulated by PCs, combined with antiangiopoietin-2 can control lung metastases [306]. In summary, many strategies can affect the growth and metastasis of tumors by targeting PCs, such as targeting metabolic enzymes, inhibiting genes, or inhibiting signaling and pathways.

PCs are important cellular components in TME and are associated with tumor angiogenesis, tumor metastasis, resistance to anti-tumor therapy, and patient mortality [283]. In terms of tumor-promoting mechanisms, it not only cooperates with ECs to regulate angiogenesis, but also promotes tumor growth and progression through various mechanisms such as regulating immune responses in TME, secreting soluble factors, and transforming into other stromal cells. Based on its tumor-promoting effect, some studies have also been carried out on the treatment of tumors by PCs, and some therapeutic strategies have seen efficacy in cell and preclinical animal experiments. However, there are some reports to the contrary, such as studies showing that the higher PC coverage in some patients, the better prognosis [307], and in some cases targeted PCs can even promote tumor metastasis [308], suggesting that the regulatory role of PCs in TME is far more complex than we think. In addition, due to the lack of effective methods to isolate human PCs, most in vitro PCs studies are carried out with bovine/mouse periretinal cells or mouse peribrain cells [288], and there is a lack of cell models to study the biology of human PCs, this difference will affect our understanding of PCs in human tumors, so the research on PCs still needs to go a lot.

## Conclusions and perspectives

TASCs are mainly composed of CAFs, MSCs, CAAs, TECs, and PCs, and the source of which can be formed by the recruitment of adjacent endogenous stromal cells or transdifferentiation from cancer cells or other stromal cells in TME. TASCs together with immune cells and tumor cells constitute the cellular components of TME, of which TASCs account for about 50% of the total number of tumor tissue cells [309]. Complex signals have been established among tumor cells, immune cells, and stromal cells, and each TASCs can communicate with components of the microenvironment in a paracrine manner through cytokines and mediators, or in a cell-cell interaction, thereby participating in the entire process of tumorigenesis and development. For example, stromal cells play a role in inhibiting tumors in the early stage of tumor development or in normal tissues, but as the tumor progresses stromal cells in TME are edited by cancer cells and other components to form a phenotype that promotes tumor development. TASCs that obtain a tumor promoting phenotype can promote the occurrence, development, and resistance to tumor therapy by secreting various factors and exosomes, participating in tumor angiogenesis and tumor metabolism, participating in tumor immune escape, and regulating extracellular matrix. Based on this, targeting TASCs is also a strategy for cancer treatment, including but not limited to preventing stromal cells from entering TME, reducing tumor-promoting stromal cells and the physical barriers they formed, reprogramming tumor promoting stromal cells into tumor inhibiting stromal cells or normal cells, and blocking communication between stromal cells and cancer cells.

CAFs are the most abundant stromal cells in the TMEs of a variety of tumors and also the most studied and the most in-depth stromal cells [37]. It can be formed by transdifferentiation of normal fibroblasts in tumor tissue, or by transformation of other TASCs and immune cells. CAFs can be divided into multiple subtypes and different subtypes have different functions and spatial distribution, but most phenotypes have pro-tumor effects, they can release growth factors, chemokines, exosomes, and metabolites, thereby reshaping the extracellular matrix, promoting cancer cell growth, promoting angiogenesis, and influencing tumor cell resistance to antitumor therapy. The strategy of using CAFs as target cells to treat tumors include direct targeting of CAFs and indirect targeting of other therapies that affect CAFs. In addition, due to the presence of both tumor-promoting and tumor-inhibiting CAF subpopulations in TME, reducing the tumor-promoting CAFs subpopulations in TME and increasing the tumor-inhibiting CAFs subpopulations can make targeted CAFs more accurate in treating tumors. Some of these therapeutic strategies have seen anti-tumor effects in preclinical experiments. However, few clinical trials targeting CAFs to treat tumors have been carried out, and no specific inhibitor of CAFs has been approved for tumor treatment so far. However, since CAFs represent the majority of cells in the tumor stroma and most CAFs have pro-tumor properties, targeting CAFs to treat tumors will become a promising and important strategy for treating tumors.

In addition, MSCs are another large class of stromal cells in TME, which can be isolated from various tissues such as bone marrow, adipose tissue, umbilical cord, or induced by pluripotent stem cells. MSCs can migrate to the tumor site under the action of a variety of cytokines and chemokines in TME, and assume different roles at different stages of tumor development. For example, in vitro cell studies on MSCs have shown that MSCs have the effect of inhibiting tumorigenesis and growth, but MSCs nesting to the tumor site are “re-educated” by tumor cells and other cells in TME, which makes MSCs have the effect of promoting tumorigenesis, development, and metastasis. For example, MSCs can promote the occurrence and development of tumors through mechanisms such as cell-to-cell contact, secretion of biomolecules, enhancement of angiogenesis, inhibition of immune cell activity, or conversion to CAFs. That is to say, endogenous MSCs are involved in the occurrence and development of tumors, but exogenous MSCs, such as administration of MSCs have less effect on promoting tumor growth. Based on this and the characteristic of tumor homing ability of MSCs, MSCs can be used as carriers for the treatment of tumors with a variety of anti-tumor substances. In addition, MSCs also contribute to hematopoietic reconstruction after hematopoietic stem cell transplantation and reduce the side effects caused by anti-tumor therapy. A large number of clinical trials have been conducted on the application of MSCs in tumors, and some trial results have also shown that the use of MSCs can bring benefits to cancer patients. Therefore, using some characteristics of mesenchymal stem cells to treat tumors is a promising tumor treatment strategy.

CAAs are also an important component of cellular composition in TME, which can be derived from MSCs or undifferentiated adipocyte precursors in adipose tissue matrix, and a small portion of CAAs can also be derived from CSCs. CAAs can participate in tumor progression, metastasis, and resistance to antitumor therapy by secreting cytokines, adipokines, providing metabolites, and transdifferentiation into other cells. Therefore, targeting CAA is also a means of treating tumors, and specific strategies include converting CAAs into normal fat cells, inhibiting related bioactive molecules and exosomes, etc. But so far, there have been no approved drugs or registered clinical trials targeting CAA for tumor treatment. However, based on the tumor-promoting effect of CAAs, targeting CAA will surely become an option for tumor treatment.

In addition, the tumor vascular system is essential in multiple stages of tumor growth and metastasis. The tumor vascular system formed by the interaction between TECs and PCs in TME participates in tumor oxygen, nutrient supply, waste removal and provides a channel for tumor metastasis, and the formed vascular system is chaotic, unhierarchical, abnormally dilated, high penetration and low perfusion, which affects the delivery of drugs to the interior of the tumor and becomes a problem for the treatment of tumors. Furthermore, TECs and PCs not only support tumor growth and metastasis through the formation of blood vessels, but also participate in tumor occurrence, development, metastasis, and resistance to anti-tumor therapy through various pathways such as secretion of soluble factors and extracellular vesicles, inhibition of anti-tumor immune responses, and transformation into other stromal cells. which makes TECs become an important target cell of anti-tumor therapy. Among the many strategies based on TECs for the treatment of tumors, anti-angiogenic therapy is the most studied, and it is also the only drug approved for clinical application in the strategy of targeting stromal cells for tumor treatment, and there are currently more than 10 anti-angiogenic drugs approved by FDA or NMPA, which has become a mature and effective anti-cancer treatment. In addition, TECs can also be inhibited by targeting certain targets and pathways of EC, but most of these strategies are in the preclinical trial stage.

At the same time, as an essential component of TME, PCs not only coordinate with ECs to regulate angiogenesis and promote tumor growth and metastasis, but also promote tumor growth and progression through mechanisms such as regulating immune responses in TME, secreting soluble factors, and transforming into other stromal cells. Based on this, treatments targeting PCs can also affect tumor growth and metastasis, and specific strategies including acting on metabolism-related enzymes, inhibiting certain genes or inhibiting signals, pathways, etc, and some of these treatment strategies have already shown efficacy in cellular and preclinical animal experiments. However, few clinical trials have been carried out. A clinical trial registered in the clinicaltrials.gov database on targeted PC for the treatment of tumors is “Nintedanib (BIBF1120) in Thyroid Cancer” (NCT01788982), in which nintedanib is a triple angiogenesis inhibitor that may act not only on endothelial cells but also on pericyte and smooth muscle cells. The primary endpoint of this clinical trial is progression free survival (PFS). With the in-depth research on PCs, we also look forward to the development of various clinical trials targeting PC for tumor treatment.

It is possible to target TASCs to treat tumors based on the influence of TASCs on tumorigenesis and development. However, since there are no specific markers for each stromal cell in TASCs, until now the other strategies targeting stromal cells for tumor treatment have not been approved in clinical in addition to anti-angiogenic drugs targeting TECs. But it is not enough to not take into account the strategy of anti-TASCs for tumor treatment based on the influence of stromal cells on tumorigenesis, development and treatment in TME. At the same time, the use of single-cell RNA sequencing (scRNA-seq), which not only improves the resolution of stromal cells in healthy and disease tissues, but also finds that each stromal cell can be divided into a variety of subtypes. Further research on different subtypes and strategies based on subtype therapy for tumors will inevitably improve the accuracy and effectiveness of tumor treatment. Therefore, targeting TASCs to treat tumors will become an important direction for tumor treatment.

### Reporting summary

Further information on research design is available in the Nature Research Reporting Summary linked to this article.

## Supplementary information

Reporting Summary
